# Ex vivo, in vivo and *in silico* studies of corneal biomechanics: a systematic review

**DOI:** 10.1007/s13246-024-01403-2

**Published:** 2024-04-10

**Authors:** Maria Vittoria Mascolini, Ilaria Toniolo, Emanuele Luigi Carniel, Chiara Giulia Fontanella

**Affiliations:** 1 Department of Industrial Engineering, University of Padova, Padova, Italy; 2 Centre for Mechanics of Biological Materials, University of Padova, Padova, Italy

**Keywords:** Cornea, Experimental tests, Finite element model, Human cornea, Computational modelling

## Abstract

Healthy cornea guarantees the refractive power of the eye and the protection of the inner components, but injury, trauma or pathology may impair the tissue shape and/or structural organization and therefore its material properties, compromising its functionality in the ocular visual process. It turns out that biomechanical research assumes an essential role in analysing the morphology and biomechanical response of the cornea, preventing pathology occurrence, and improving/optimising treatments. In this review, *ex vivo, in vivo* and *in silico* methods for the corneal mechanical characterization are reported. Experimental techniques are distinct in testing mode (e.g., tensile, inflation tests), samples’ species (human or animal), shape and condition (e.g., healthy, treated), preservation methods, setup and test protocol (e.g., preconditioning, strain rate). The meaningful results reported in the pertinent literature are discussed, analysing differences, key features and weaknesses of the methodologies adopted. In addition, numerical techniques based on the finite element method are reported, incorporating the essential steps for the development of corneal models, such as geometry, material characterization and boundary conditions, and their application in the research field to extend the experimental results by including further relevant aspects and in the clinical field for diagnostic procedure, treatment and planning surgery. This review aims to analyse the state-of-art of the bioengineering techniques developed over the years to study the corneal biomechanics, highlighting their potentiality to improve diagnosis, treatment and healing process of the corneal tissue, and, at the same, pointing out the current limits in the experimental equipment and numerical tools that are not able to fully characterize in vivo corneal tissues non-invasively and discourage the use of finite element models in daily clinical practice for surgical planning.

## Introduction

The cornea is an in vivo pressurized dome-like tissue, constantly stimulated outwards by a net internal pressure, defined as intraocular pressure (IOP), due to the aqueous humor inside the anterior chamber of the eye. Normal IOP values range from 13 to 21 mmHg [1, 2] with cyclic fluctuations of 2–4 mmHg during the day [1], while increases may occur for multiple causes, from simpler conditions like the respiration to more severe reasons like accidental impact on the ocular surface or occurrence of ocular pathology such as glaucoma [2, 3]. Both the thickness and structural organization ensure the corneal mechanical properties needed to guarantee its functionality as protection for the inner ocular components and major refractive power of the ocular system. It has been shown that corneal biomechanics is impaired in myopic patients [4, 5], as well as in patients affected by other ocular diseases (such as keratoconus [6, 7], glaucoma [8], dry eye disease [9]). Therefore, assessing the mechanical behaviour of the cornea is clinically crucial for understanding first of all the physiological behaviour of the tissue and, in this respect, for diagnosing potential pathologies responsible for the weaking, thinning or curvature variation, and quantifying the success of refractive surgery and therapeutic treatments.

Ex vivo uniaxial tensile testing is the most spread method [10–12] to study corneal biomechanics, consisting in cutting a rectangular corneal specimen along a specific anatomical direction, usually including a portion of the sclera used to grip the specimen by the mechanical clamps of a tensile machine. The sample is generally stretched with a defined velocity of elongation, and the resulting force is measured and converted to the stress as the ratio between the load and the original cross-section area of the sample. There are many implemented protocols, which can also quantify the time-dependent proprieties (i.e., stress relaxation [12–18] and cyclic tensile loadings at different strain rates [19, 20] to reproduce slow actions due to IOP variation with posture change and fast actions as accidental impacts [20]) and failure behavior [10, 11, 15–18, 21–26]. Inflation tests were used for structural analysis to overcome the limitations of tensile tests, such as cutting of the samples which may alter the corneal microstructural arrangements and flattening of the corneal curvature, and to propose a more physiological-like environment, assessing more reliably the corneal mechanical properties. They consist of inflating corneal buttons or eye globes by using artificial chambers or ad hoc developed instruments, at an imposed pressure or volume. The main protocols adopted were loading-unloading tests, by increasing-decreasing pressure, and creep tests, where the pressure was maintained constant [12, 27, 28]. By ex vivo tests, the mechanical properties of the corneas, such as Young’s Modulus, tensile strength, and apical displacement, were measured in healthy conditions but also in samples lesioned by means of alkali solution simulating melting ulcers [14, 24] or treated with cross-linking (CXL) by means of the combined action of riboflavin drops and UVA irradiation [14, 29–34].

Although ex vivo techniques are relatively simple and low cost and have contributed over the years to achieve important levels of the current knowledge on corneal biomechanics, the interest in direct methods to assess the in vivo mechanical behaviour is growing nowadays. Currently, there are mainly two clinical instruments available, the Ocular Response Analyzer (ORA) and the Corvis ST [7], both studying the dynamic applanation of the cornea in response to an air puff of short duration on the tissue external surface. They provide output parameters related to the characteristics of the puff, which are different from each other and with the ex vivo parameters, so direct comparisons in the measurement of cornea Young’s Modulus cannot be performed.

However, starting from the experimental-derived evidence, novel promising approaches can be introduced by computational modelling, in particular finite element methods (FEMs), that are applied successfully in several surgical fields [35–39] and are spreading also in ophthalmic research [40, 41]. Reliable FEMs require to be validated by means of strong comparisons with physical reality, by implementing healthy and pathological conditions, simulating the experimental set-ups and obtaining numerical results able to correctly report what has happened in clinics or labs. This *in silico* methodology is complex, time-consuming, expensive [42] and has to be performed meticulously but, once it is completed, a computational clinical tool can be exploited in several ways: quantifying numerically the changes in IOP better and not invasively, improving corrective surgical procedures, optimizing preclinical patient evaluation, personalizing treatments and predicting alterations anticipating diagnosis.

Therefore, given the several samples’ species, conservation and cutting methods, numerous setups and the variety of testing machines, this review was aimed at providing a summary of the recent ex vivo and in vivo experimental studies, highlighting the differences among the works, the results and the limitations of these approaches. At the same time, this work was intended to propose the high-impact potentialities of the computational approach if properly validated with experimental methods, pointing out the extreme variability in terms of geometry, finite element discretization, material formulations and boundary conditions that made them appropriate for basic research, but too immature for surgery and diagnostics.

## Methods

### Study design

A systematic review was performed using the search engines PUBMED and SCOPUS and the search terms “([{cornea} AND {mechanic* OR stiff*} AND {properties} AND {tensile OR inflation OR indentation OR in vivo OR FEM OR simulation}]).” Searches were limited to the English language and to year of publication from 2001 to 2023 for ex vivo and in vivo studies and from 2014 to 2023 for *in silico* studies. The latest search was performed on 2th September 2023. PICO framework helped in developing studies evaluation:

P (population/problem): assessing corneal biomechanics.

I (intervention or exposure): experimental and numerical techniques.

C (comparison): ex vivo, in vivo and *in silico* methods.

O (outcome): testing protocols, instrumentations, samples preparation, mechanical quantities, pro and cons of approaches.

### Eligibility criteria

For this review, eligibility criteria included studies which employed ex vivo, in vivo and *in silico* techniques to mechanically characterize the human cornea or the porcine cornea, recognized in literature as the most human-like model. Studies which did not report details on the methods used for mechanical assessment or focused only on different animal species were excluded, even though some recent publications started reconsidered rabbit model [43–45] maybe due to the easier collection and availability. By the inspection of the titles and abstracts, 68 articles met the inclusion criteria and were reviewed by reviewers (M.V.M and I.T.): 32 studies on ex vivo techniques, 17 studies for in vivo techniques, 18 studies on in *silico* techniques.

### Data extraction

For each study, first of all the authors’ name, year of publication, tissue species and type of testing were recorded. Subsequently, based on the nature of the test, the following information were specifically considered: for ex vivo studies, the geometry of corneal specimen and its measurement, the corneal populations, the test protocol, the experimental setup and the mechanical parameters obtained; for in vivo studies the principal methodologies focusing on instruments currently used in the clinic; for *in silico* studies, the goal, the geometry of the model, the technique for FE discretization, the material formulation, the boundary condition and the software used.

## Methods of corneal mechanical characterization

### Ex vivo studies

A standard and not-invasive procedure to collect the biomechanical properties of human and animal corneas through in vivo tests is still not been implemented. The best compromise consists of using human wastes derived from refractive surgery or animal eye cornea derived from local abattoirs, and in very few cases, human corneas obtained from eye banks. The latter is very complicated because of the shortage in having samples suitable for transplants, preferring to allocate the samples for that aim instead of for scientific testing.

In this paragraph, the existing studies on corneal biomechanics investigated by means of ex vivo uniaxial tensile testing (Table 1), inflation test (Table 2) and other mechanical tests (Table 3) are proposed, classifying them in terms of corneal species (human or porcine), typology of the test (such as, tensile up to failure, stress-relaxation or loading/unloading cycles), corneal populations analysed (such as related to the specimen orientation and/or experimental condition), measures of corneal strips, mechanical protocol and experimental setup, including the tensile machine, the corneal preservation, hydration and treatment methods. All the samples considered in the studies were extracted in healthy corneal conditions without any active pathological condition affecting the tissue. Animal corneas were generally obtained from pigs aged between 4 and 6 months from slaughterhouse [11–17, 19, 20, 23–26, 29, 30, 32], while human corneal specimens were harvested from cadaver donor eyes as full-thickness samples [12, 13, 22, 29, 30], or removed as stromal lenticules from the central cornea during surgery [5, 10, 21, 27, 46].

**Table 1:** Ex vivo tensile testing on cornea strips

Study	Origin	Test	Population	Sample characteristics	Test protocol	Setup	Results
[10]	*SMILE* refractive surgery	Tensile up to failure	H stromal lenticules removed from cornea during the SMILE surgery cut along NT and SI directions (4 males, 6 females, age 22.00 ± 5.00 years)	No. of strips: 20 Length: 6.00 mm Width: 1.20 mm	Samples preconditioned by 3 displacement cycles between 0 and 0.50 mm and then loaded up to failure with elongation rate of 0.6 mm min^-1^	Lenticules were preserved in medium Eusol-C (Alchimia, Padova, Italy) below 4 °C in refrigerator for less than 24 h. The tests were performed IBTC-50 testing system (Care Measurement & Control Co., Ltd., Tianjin, China) in water bath apparatus filled with PSS at room temperature of 25℃	For strain less than 5%, Young’s Modulus 1.30 ± 0.51 MPa in NT direction and 1.14 ± 0.28 MPa in SI direction. Tensile strength 14.05 ± 1.95 MPa in SI direction and 13.25 ± 2.16 MPa in NT direction
[21]		Tensile up to failure	H stromal lenticules removed from cornea during the SMILE surgery cut along NT and SI directions (11 males, 21 females, age 21.11 ± 3.24 years)	No. of strips: 64 Length: 6.5 mm Width: 1 mm	The distance between the two clamps was 1.5 mm. The samples were preconditioned with three loading/unloading cycles and then stretched up to failure with deformation rate of 0.01 mm s^-1^	Lenticules were preserved in medium Eusol-C (Alchimia, Padova, Italy) below 4 °C in refrigerator for less than 24 h. Tests were performed using an IBTC-50 testing system (Tianjin Care Measure and Control Co., Ltd., Tianjin, China) in a laboratory water bath filled with PSS at room temperature	At stress 0.02 MPa, Young’s Modulus 1.75 ± 0.45 MPa in NT direction and 1.84 ± 0.64 MPa in SI direction. For strain less than 20%, low-strain tangent modulus 1.17 ± 0.43 MPa in NT direction and 1.32 ± 0.50 MPa in SI direction. For strain between 35% and 55%, high-strain tangent modulus 43.59 ± 7.96 MPa in NT direction and 51.26 ± 8.23 MPa in SI direction
[5]		Tensile	H stromal lenticules removed from cornea during the SMILE surgery (7 males, 15 females, age 23.96 ± 5.27 years)	No. of strips: 24 Length: 6.60 mm Width: 1.00 mm	Specimens underwent 2 loading/ unloading cycles for preconditioning. The rate of clamp displacement was 0.05 mm s^-1^ and the maximal force was 0.25 N	Lenticules were preserved in medium Eusol-C (Alchimia, Padova, Italy) at 4 °C before testing. A custom-built tensile testing system, composed by 1 N load cell capable (ELFST3E-2 L, Entran Devices Inc., Fairfield, NJ, United States), and platform driven by a stepper motor, combined with customized SD-OCT imaging subsystem (superluminescent diode with a central wavelength of 840 nm, a bandwidth of 45 nm and an output power of 4.5 mW) was used. Samples were bathed in PSS during testing	Low-strain tangent modulus 0.204 ± 0.189 MPa and high-strain tangent modulus 5.114 ± 1.958 MPa
[46]		Tensile	H stromal lenticules removed from the central cornea during the SMILE surgery (16 males, 21 females, age 25.14 ± 6.74 years)	No. of strips: 63 Length: 6–6.5 mm Width: 1 mm CCT: 584.16 ± 24.14 μm	Tensile force applied with a speed of 0.01 mm s^-1^ uniaxial tension. The stress corresponding to less than 5% of the strain was analyzed	Lenticules were preserved in medium Eusol-C (Alchimia, Padova, Italy) below 4 °C in refrigerator for less than 24 h. A mechanical test system (ITBC-50, Kyle Measurement and Control Test System Co., Ltd.) was used, samples were mounted and kept wet with PSS	At stress of 0.02 MPa, Young’s Modulus 2.45 ± 1.72 MPa
[22]	*Donors*	Tensile up to failure	H complete corneas from donors (average age 28 years)	No. of strips: 30 Length: 12 mm Width: 4 mm CCT: 600 ± 60 μm	Strips stretched until the fracture point	Tests were conducted using Instron 5566 (± 50 N load cell). Sclera tissue at the end cornea strip was used for the attachment to the mechanical grips	8 parameters of the AFHVE model obtained by utilizing the coupled FE/OPT algorithm: τ of 180, k of 0.39, g of 0.28, *k* of 0.17, *k2* of 238, *k1* of 240 kPa, D of 4760 kPa, C10 of 289 kPa
[13]		Stress relaxation at different stress levels	H donor (age 75.6 ± 6.1 years) corneas unsuitable for transplantation due to low endothelial cell counts and P corneas cut along SI direction	No. of strips: 12 (H), 10 (P) Length: 10 mm Width: 3 mm Scleral ring: 2 mm	Samples subjected to strain rate of 10% min^-1^. When the load reached 4 N, loading stopped and the specimen maintained at a constant length for 20 min. The load was then increased to 8 N, followed by 12 N, and at each load level, the length maintained for 20 min	Before testing, a perspex tube was placed around the specimen and filled with Eusol-C to maintain stromal hydration during the tests. Testing was performed on an Instron 3366 machine equipped with a 50 N capacity load cell	Average stress reductions 27.7 ± 5.6% after 400 s, 30.5 ± 5.7% after 800 s and 32.0 ± 5.7% after 1200 s for H corneas stretched with the length reached under load of respectively 4, 8 and 12 N, while 49.2 ± 8.3%, 55.6 ± 8.2% and 59.2 ± 8.1% as corresponding average stress reductions for P corneas
[12]		Stress relaxation and tensile up to failure	H corneal rings left over from corneal transplant operations and P corneas	No. of strips: 19 (H), 19 (P) Length: 12–16 mm Width: 2.5 mm Mean thickness: 1 mm	Samples were preconditioned by 3 load/unload cycles under 10 mm min^-1^ velocity. On 10 H and 10 P corneas, a load was applied at a 10 mm min^-1^ velocity until failure. On 9 H and 9 P corneas, stress relaxation was performed with 250 mm min^-1^ elongation speed and stretch ratio 1.5 maintained for 1000 s	As the H corneas were placed in Optisol and stored below 4 °C for a few days before the transplant operation, the same was performed for P corneas. Tests were conducted using an Instron apparatus at room temperature and an ultrasound moistener was used to keep the specimen moist	Average tensile strength 3.81 ± 0.40 MPa for H corneas and 3.70 ± 0.24 MPa for P corneas. By describing stress-relaxation behaviour by the slope K of the normalized relaxation curve in function of a log time and the value P at the end of relaxation: for stretch ratio of 1.5, K and P for relaxation of 1000 s respectively 0.0165 ± 0.0024 and 85.6 ± 1.5 for H corneas, while 0.0553 ± 0.0069 and 64.6 ± 3.3 for P corneas
[30]	*CXL treatment on donors*	Tensile up different strain levels	H corneas from eyes enucleated because of damage to other ocular tissues and P corneas, cut along SI direction and distinct in untreated and CXL-treated groups	No. of stirps: 5 (H), 20 (P) Length: 14 mm Width: 4 mm (H), 5 mm (P) Scleral ring: 2 mm CCT: 550 ± 40 μm (H), 850 ± 70 μm (P)	A prestress of 5 × 103 Pa was used, requiring a force of 10 mN in H and 20 mN in P corneas. The strain was then increased linearly with a velocity of 1.5 mm min^–1^, and the stress was measured up to 2 × 10^5^ Pa	Before treatment, the corneal epithelium was scraped. 0.1% riboflavin photosensitizer solution was dropped on the treated strips and 20% dextran solution on the control strips at 5-min intervals. UVA irradiation (370 nm) was applied using 2 double UVA diodes (irradiance 3 mW/cm^2^ at 1.0 cm distance from the cornea for 30 min). Strips were clamped between the jaws of a commercially available microcomputer-controlled biomaterial tester (Minimat, Rheometric Scientific GmbH)	At 4% strain, average Young’s Modulus 0.8 MPa and 1.4 MPa respectively in untreated and CXL treated P corneas, and 0.8 MPa and 3.0 MPa respectively in un-treated and CXL treated H corneas. At 6% strain, average Young’s Modulus 1.5 MPa and 2.7 MPa respectively in untreated and CXL treated P corneas, and 1.3 MPa and 5.9 MPa respectively in un-treated and CXL treated H corneas. At 8% strain, average Young’s Modulus 2.6 MPa and 5.3 MPa respectively in untreated and CXL treated P corneas, and 2.2 MPa and 11.8 MPa respectively in un-treated and CXL treated H corneas
[29]		Tensile	H donors (age 65–84 years) corneas not suitable for transplantation and P corneas, cut along SI direction and distinct in untreated and CXL-treated groups	No. of strips: 10 (H), 40 (P) Length: 7 mm Width: 5 mm Thickness: 200 μm	The distance between the clamps was 3 mm, the strain rate was 2 mm min^-1^, and the prestress 10^4^ N/m^2^	The epithelium of 20 P and 5 H eyes was removed. 5 min before the irradiation, 0.1% riboflavin photosensitizer solution was applied repeatedly to the cornea at 5-minute intervals during the whole treatment. The UVA light (370 nm) was applied using 2 UVA diodes (irradiance of 3 mW/cm^2^ at 2 cm distance from the cornea for 30 min). In other eyes, only the epithelium was removed but no treatment was performed (control). After irradiation, the intraocular pressure was increased to 20 mmHg by injecting PSS through the optic nerve for good cutting conditions. 2 flaps were cut with the microkeratome from each eye. Samples were clamped between the specimen support of a biomaterial tester (Minimat, Rheometric Scientific GmbH)	At 5% strain, average Young’s Modulus 3.6 MPa and 1.3 MPa respectively for anterior and posterior untreated H samples, while 6.0 MPa and 1.0 MPa respectively for anterior and posterior treated H samples. At 5% strain, average Young’s Modulus 2.9 MPa and 2.8 MPa respectively for anterior and posterior untreated P corneas, while 6.3 MPa and 2.7 MPa respectively for anterior and posterior treated P samples
[23]	*Animal model*	Tensile up to failure	P corneas cut along SI direction	No. of strips: 10 Length: 12 mm Width: 5 mm	Samples stretched up to failure with strain rate of 1 mm min^-1^	Samples were coated with mineral oil to minimize hydration before and during the test. Tests were performed on Instron machine (50 N capacity load cell)	Highly nonlinear behaviour with load –elongation relationship linear up to 16 N and rupture between 23 and 26 N. The average overestimation of stiffness due to tensile test rather than inflation reduced from about 32 to 5% by the novel introduced procedure
[11]		Tensile up to failure under different strain rates	P corneas from the same animal cut along NT, SI and 45° or 135° diagonal directions	No. of strips: 108 Length: 12 mm Width: 3 mm CCT: 955 ± 24 μm Sides thickness: 1074 ± 21 μm Scleral ring: 3–4 mm	Samples were subjected to uniaxial tension starting with three load cycles between 0 and 10 N to condition the specimen, followed by loading to failure. Samples were subjected to three elongation rates, 0.1, 1 and 25 mm min^-1^ (approximately 0.8, 8.3 and 210% min^-1^ )	Corneas were placed in preservation medium Eusol C (Alchima, Padova, Italy) for a maximum of 4 h. The specimens were connected to mechanical clamps with rough surfaces to prevent slippage. Before the start of test, a perspex tube was placed around the specimen and filled with Eusol C to continue maintaining hydration during the tests. Testing was performed at room temperature, 21 °C, on an Instron 3366 materials testing machine (Instron, Norwood, MA) equipped with a 5 0 N capacity load cell	Small increases of the stiffness by increasing the strain rate. In NT direction, for 0.01 MPa stress, Young’s Modulus 0.379 ± 0.054 MPa, 0.382 ± 0.030 MPa and 0.423 ± 0.031 MPa respectively for 0.8, 8.3 and 210% min^-1^; for 0.03 MPa stress, 1.124 ± 0.155 MPa, 1.133 ± 0.090 MPa and 1.252 ± 0.090 MPa respectively for 0.8, 8.3 and 210% min^-1^. In SI direction, for 0.01 MPa stress, Young’s Modulus 0.343 ± 0.064 MPa, 0.349 ± 0.053 MPa and 0.397 ± 0.056 MPa respectively for 0.8, 8.3 and 210% min^-1^; for 0.03 MPa stress, 1.016 ± 0.183 MPa, 1.036 ± 0.155 MPa and 1.181 ± 0.164 MPa respectively for 0.8, 8.3 and 210% min^-1^. In diagonal direction, for 0.01 MPa stress, Young’s Modulus 0.374 ± 0.022 MPa, 0.426 ± 0.103 MPa and 0.427 ± 0.037 MPa respectively for 0.8, 8.3 and 210% min^-1^; for 0.03 MPa stress, 1.109 ± 0.065 MPa, 1.249 ± 0.302 MPa and 1.264 ± 0.111 MPa respectively for 0.8, 8.3 and 210% min^-1^
[25]		Tensile up to failure under different strain rates	P corneas from different animals cut along NT and SI direction and corneas from the same animal cut along NT and SI directions	No. of strips: 84 CCT: 0.8–0.9 mm Length: 12 mm	Random samples were preconditioned through 3 load/unload cycles with a single strain rate for each sample (0.1, 1, 3, 10 or 50 mm min^-1^) and 1.4 N maximum load applied, and then stretched until failure. Other samples were preconditioned by 1–4 cycles with different strain rates for each sample (0.1, 1, 3, 5, 10, 50 mm min^-1^) and 14 N maximum load applied, and then loaded until failure	Tests were conducted at room temperature on an Instron 3366 materials testing machine (load cell capacity 50 N). To avoid swelling, tissue corneal rings were put into PSS for 2–3 min to drain out water	On average the SI specimens 34% stiffer and with higher rupture strength than the NT ones
[15]		Tensile up to failure, stress- relaxation and test for transversal contraction	P corneas cut along NT and SI directions	No. of strips: 47 Length: 20 mm Width: 3 mm	Samples were stretched in 20 cycles at constant strain rate (range 0.01–0.1 s^-1^ of the original length) with axial load of 50mN. To reproduce physiological stress state, samples were subjected to an initial axial load of 50 mN. The stress-relaxation test was performed with constant strain levels of 2, 4, 8 and 10% applied for 50, 100, 1000, 2000 s, respectively and then samples were stretched up to failure. For transversal contraction test, samples were preconditioned and then relaxation tests at 5, 10 and 15% strain levels were performed with relaxation times respectively of 100, 1000 and 3000 s	Stress-relaxation and failure tests were performed using a uniaxial system (MTS SynergieEden Prairie, MN USA) with 100 N capacity load cell. During the tests, samples were immersed in a hydration chamber filled with Eusol-C at room temperature to prevent dehydration. Tests for transversal contraction was performed by electromagnetic system ELF3200 (Bose Corporation,ElectroForce Systems Group, Eden Prairie, Minnesota, USA) with 22 N capacity load cell, equipped with a transparent hydration chamber to take pictures of the sample. External surface of the sample marked by permanent ink with 2 vertical and 2 horizontal reference lines. Distance between the reference lines at different strain levels measured from images taken with a Nikon DS-5 camera connected to a Nikon SMZ800 stereo-microscope	Young’s Modulus 3.193 ± 1.589 MPa for strain range up to 4% and 41.806 ± 10.920 MPa for strain range 6 − 12%. Yield stress 3.837 ± 1.312 MPa, yield strain 15.4 ± 2.4%, failure stress 4.763 ± 1.251 MPa and failure strain 19.2 ± 2.3%. SI strips characterized by higher stiffness for strains higher than 0.06, higher yield stress and higher failure stress with respect to NT strips. For strain levels higher than 12%, SI specimens 21% more rigid with 17% higher failure stress than the NT ones. For 2% strain constant for 50 s, relaxation stress percentage of 16.1% from a peak stress of 0.059 ± 0.039 MPa with a time constant of 6.165 ± 1.649 s, while for 10% strain constant for 2000 s respectively the corresponding values 0.322 ± 0.049%, 1.654 ± 0.611 MPa and 471.101 ± 75.229 s
[16]		Tensile up to failure and stress relaxation	P corneas	No. of strips: 42 Length: 12 mm Width: 4 mm	Samples stretched until failure by speed of 20 mm min^-1^. For stress-relaxation tests, strips stretched up to 1.25 times their length at 120 mm min^-1^ with 1000 s relaxation time	During the test, strips are clamped with biological material holder	On average, the initial and the last stress respectively as 2.33 MPa and 0.88 MPa. Stress-relaxation curve fitted by the modified Maxwell viscoelastic model (relaxation modulus E1: 0.69 MPa, E2: 0.43 MPa, E3: 0.31 MPa, E4: 0.40 MPa, E5: 0.43 MPa; relaxation time τ1: 8.83 s, τ2: 65.33 s, τ3: 876.93 s and τ4: 2.84 × 10^3^ s)
[17]		Tensile up to failure, stress-relaxation, trouser shear test	P corneas cut along SI direction	No. of strips: 24 (normal), 12 (with notch) Length: 10 mm (normal), 4 mm (with notch) Width: 5 mm (normal), 10 mm (notch)	Samples were stretched at different rates: 3, 30 and 300 mm min^-1^, until failure. For stress-relaxation testing, samples were strained to 2- and 4-mm levels in 5 s and held at fixed displacement for 120 s. For trouser tear test, samples were stretched at 3, 30 and 300 mm min^-1^ extension rates, by stopping the test before the crack had fully propagated along the specimen length	All specimens were submerged in PBS for 2 h prior to testing. Small pieces of sand paper attached to the ends of specimens for gripping. Mechanical tests were performed with a universal testing machine (model 5544, Instron, Canton, MA) equipped with a 500 N load cell, at room temperature. After mounting onto the machine, all corneas were sprayed with PBS to minimize any dehydration effects	Young’s Modulus 9.59 ± 1.28 MPa, 10.29 ± 0.85 MPa and 9.82 ± 1.39 MPa respectively for 3, 30 and 300 mm min^-1^. Equilibrium normalized load at 2 and 4 mm respectively of 0.19 ± 0.02 and 0.41 ± 0.02
[26]		Tensile up to failure	P corneas cut along NT, SI and 45° and 125° diagonal directions, with incisions along thickness at depths of 300 and 600 μm (anterior, central and posterior layers)	No. of strips; 72 Length: 10 mm Width: 3 mm CCT: 979 ± 37 μm Scleral ring: 3–4 mm	Samples were preconditioned by 5 load/unload cycles (range 0.01–0.1 of the original length) under a 3 mm min^-1^ loading velocity. To reproduce the physiological stress state, an initial axial load of 50 mN was applied. Then, specimens were loaded up to failure under a 1 mm min^-1^ stretching velocity just 300 s after their recovery	The corneal epithelium was removed, then the whole eyeballs were submerged in 20% Dextran solution at most 4 h in a refrigerator at 4 °C to reinstate the corneal thickness to the physiological level. Tests were carried out on an Instron 5848 materials testing machine (Instron, Norwood, MA) equipped with a 5 N capacity load cell at room temperature (22 °C). The specimens were attached to the mechanical clamps with 320-grit sandpaper to avoid slippage. An ultrasound moistener was used to keep the specimen moist	Young’s Moduli in different orientations equal to each other: at stress of 0.03 MPa, for the anterior layer 2.869 ± 0.584 MPa, 2.484 ± 0.740 MPa and 2.706 ± 0.707 MPa respectively in SI, NT and diagonal directions; for the central layer 2.333 ± 0.337 MPa, 2.098 ± 0.536 MPa and 2.071 ± 0.584 MPa respectively in SI, NT and diagonal directions; for the posterior layer, 1.640 ± 0.331 MPa, 1.746 ± 0.386 MPa and 1.415 ± 0.228 MPa respectively in SI, NT and diagonal directions. Young’s Modulus decreased gradually along the depth: at stress of 0.03 MPa, 2.484 ± 0.740 MPa, 2.098 ± 0.536 MPa and 1.746 ± 0.386 MPa respectively for anterior, central and posterior layers in NT direction, 2.869 ± 0.584 MPa, 2.333 ± 0.337 MPa and 1.640 ± 0.331 MPa respectively for anterior, central and posterior layers in SI direction and 2.706 ± 0.707 MPa, 2.071 ± 0.584 MPa and 1.415 ± 0.228 MPa respectively for anterior, central and posterior layers in diagonal direction
[19]		Loading-unloading cycles under different strain levels	P stromal corneas cut along NT, SI and diagonal (± 45° from horizontal axis) directions, with each direction considered at depth of 100, 350 and 650 μm	No. of strips: 120 Length: 8 mm Width: 3.5 mm Thickness: 150 μm	Strips prestretched with a 20 mN force and then loaded with 6 cycles at a 0.75% s^-1^ strain rate (last cycle recorded for analysis) for different levels of strains (6, 8, 10, 12%)	The entire eyeball was placed in a custom pressurization device to prevent movement during corneal strip cutting with the Femto LDV Z8 Ziemer femtosecond laser (Ziemer Ophthalmic Systems AG, Switzerland). For each eye, 3 strips cut in NT, SI, and diagonal directions at a depth of 100 μm, 350 and 600 μm. Samples were tested in a hydration preserving culture media containing 5% Dextran using the Ustretch device (CellScale, Waterloo, Canada) with BioRake attachments at room temperature	Similar stiffness at 100 and 350 μm, while lower stiffness in the most posterior layer. Most anterior layers similar across orientation, contrary to the most posterior layer with stiffer response in SI direction. By increasing the strain level, the measured force for the anterior layers resulted significantly higher than the posterior one and the differences in force for the different orientations increased
[20]		Loading-unloading cycles under different strain rates	P (aged between 4 and 6 months) corneas cut along SI direction	No. of strips: 16 Length: 12 mm Width: 4.4 mm Scleral ring: 3–4 mm CCT: 971.5 ± 75.7 μm	Specimens were subjected to an initial set of 10 loading-unloading cycles between 0 and 5 N with a low strain rate of 8%min^-1^, followed by 7 sets of 3 load cycles with different strain rates (0.8, 8, 25, 42, 83, 210 and 420% min^-1^) and 360 s as recovery period between 2 sets	After extraction, corneas were placed in a preservation medium Eusol-C (Alchima, Padova, Italy). Before tests, a perspex tube was placed around the specimen and filled with Eusol-C to maintain stromal hydration during the tests. Testing was performed at room temperature, 21 °C, on an Instron 3366 material testing machine (Instron, Norwood,MA) equipped with a 10 N capacity load cell	For P corneas, large (40.2% on average) and statistically significant increase of stiffness only by varying strain rate from 0.8 to 8% min–1, while subsequent increases led to much lower and not statistically increase in stiffness.
[24]	*Treatment on animal model*	Tensile up to failure	P control and NaOH-treated corneas cut along NT direction	No. of strips: 8 Length: 16.02 ± 1.33 mm (control), 13.12 ± 2.45 mm (treated) Width: 5.24 ± 1.67 mm (control), 5.05 ± 0.97 mm (treated) CCT: 1.56 ± 0.22 mm (control), 1.76 ± 0.21 mm (treated) Scleral ring: 2 mm	Samples pre-conditioned at 5% strain regime and then subjected to a quasi-static uniaxial tensile displacement to the rupture point at a rate of 0.2 mm s^-1^	Test machine was designed, fabricated and calibrated at the Ultrasonics Lab (University of Granada, Spain), using an Imada ZTA-500 force measurement system, connected to a PC and an automated Matlab code (accuracy ± 0.2%). Displacement was obtained through the movement of the uniaxial tensile stepper motors (precision < 5 μm). For the treated group, corneal buttons were soaked in 1.5 M NaOH for 2 min, followed by washing with water and PBS each for 2 min, independently	Young’s Modulus about 10 MPa for control and about 5 MPa for NaOH-treated corneas. Tensile strength about 2.5 MPa for control and about 1 MPa for NaOH-treated corneas
[14]		Stress relaxation	P healthy or lesioned, CXL-treated or untreated, fresh or cultured corneas cut along NT direction	No. of strips: 20 Length: 10 mm Width: 5 mm	Samples were preconditioned by 10 load/ unload cycles up to 8% strain, at a strain rate of 1%s^ −1^. Then, the sample was subject to almost-instantaneous elongation (strain rate of 800%s^ −1^) followed by stress relaxation for a time period of 400 s, increasing the strain to 8, 16, 24, 32% in consecutive elongation relaxation steps	Tests were performed at room temperature with a Bose ElectroForce® Planar Biaxial Test Bench instrument (TA Instruments, New Castle, USA), equipped with a load cell of 22 N. Lesioned corneas were obtained by using a filter paper soaked with NaOH placed for 1 min on the surface before wash the corneas with PBS solution for 60 s. Fresh corneas were tested few hours after enucleation while cultured ones after 7 days preservation into CARRY-C culture medium. For CXL treatment, isoosmolar 0.1% riboflavin drops were administered for 30 min and then corneas were UVA irradiated for 3 min with commercially available equipment (Vetuvir™, Vision Engineering Italy srl, Rome, Italy) with 30 mW/cm^2^ irradiance at 10 cm from the cornea. Samples were soaked in PSS at room temperature prior to testing, and hydrated by dropping PSS during the test	Nonlinear and time-dependent behavior influenced by the structural modifications induced in lesioned and treated populations, reducing the stiffness and modifying the behavior over time. Higher stiffness in healthy untreated fresh corneas respect to other groups. Higher relaxation times for healthy, untreated and fresh or cultured groups, while independent of both lesioned and treated corneas

*H* human; *P* porcine; *NT* nasal-temporal; *SI* superior-inferior; *CXL* Cross-linking; *CCT* Central Corneal Thickness; *PSS* physiological saline solution; *PBS* phosphate buffer saline; *SMILE* Small incision lenticule extraction; *SD-OCT* Spectral domain - optical coherence tomography; *AFHVE* anisotropic fiber-reinforced hyper-viscoelastic

**Table 2:** Ex vivo inflation testing on cornea buttons or eye globes

Study	Samples	Test protocol	Setup
[27]	H eye globe: 6	Loading-Unloading: initial measurement taken at 18 mmHg and subsequently at 6 mmHg step increases. Pressure was increased to 42 mmHg and then decreased, at 6 mmHg steps, up to 18 mmHg Creep: nearly instantaneous IOP increase from 18 to 42 mmHg, then holding the IOP stable at 42 mmHg for 10 min. The IOP was then instantaneously decreased from 42 to 18 mmHg and held stable at 18 mmHg for 10 min	A custom-built apparatus, the Ocular Biomechanics Modulator (OBM), was used to perform experiments in a monitored environment. The temperature and humidity were continuously recorded. The IOP of the eye globe was monitored by means of a water column and a pressure transducer and modified by infusing saline solution into the posterior segment of the eye through a needle by an automated pumping system. Corneal topography maps of the anterior and posterior cornea were obtained using a Scheimpflug-based Pentacam HR. The whole apparatus was computer-controlled by dedicated software written in LabView
[13]	Corneal Samples: 44 P, 49 H (23 H and 19 P loading-unloading test, 14 H and 16 P creep test)	Loading-Unloading: Three cycles of loading and unloading up to 170 mmHg were applied. Creep: Corneal specimens kept to a constant level of pressure of 15 mmHg for 20 min. The test was then repeated with increased levels of pressure in steps of 5 mmHg up to a maximum level of 40 mmHg	The corneas were mounted onto the pressure chamber of the rig using mechanical clamps and cyano-acrylate glue to provide a watertight connection along the specimens’ ring of scleral tissue. the pressure chamber was filled with saline solution and connected to a small reservoir, whose vertical movement was computer-controlled to set the pressure change rate at 37.5 mmHg min^−1^. The actual pressure in the chamber was measured using a differential pressure transducer. A laser displacement sensor and two digital cameras positioned in the plane of the corneoscleral intersection were used to continually monitor corneal displacement during the tests
[51]	Eye: 1 P, 1 H	Loading-Unloading: eyes were loaded and unloaded from 2 to 60 mmHg at a rate of 40 mmHg min^−1^, with a rest period of 1 min following each of 10 loading cycles	A fixed borosilicate glass box contains the intact globe and enables its suspension in a clear gelatine material that protects the external surface of the ocular vessel from environmental conditions, provides the eye with a support system, which restricts free-body motion, is more uniform and offers a better representation of physiologic conditions than traditional support systems, and enables an unobstructed view of the entire ocular surface from outside the glass box. A 2 × 50 mm hypodermic needle was inserted into the ocular cavity through the posterior pole, passed through the back wall of the glass box and its support system and connected through a pipe network to a motordriven syringe pump, which provided changes in the applied pressure. The deformation of the eye that resulted from changing the internal pressure by the syringe pump was measured using a system of three high-resolution, digital cameras
[32]	Corneal Samples: 17 P	Loading-Unloading: Three cycles of gradual loading and unloading pressure up to 42.66 mmHg were applied to stabilize its behaviour before considering the results in the fourth cycle	A central disk including the corneal button and a 2 mm scleral ring was removed with a pair of curved scissors. The cornea was mounted onto the pressure chamber of the test rig by using the mechanical clamps and a watertight connection of cyanoacrylate adhesive Super Glue. The pressure chamber was filled with phosphate buffer solution and connected to a small reservoir, whose vertical movement was computer-controlled to set the pressure change rate at 25.6 mmHg min^−1^
[28]	Eye: 30 P	Loading-Unloading: IOP was increased from 5 to 55 mmHg steps, and then decreased at 5-mmHg intervals. Each pressure step was held constant for 1 min	Water column was filled with 0.9% saline solution and connected to the eye globes to change the IOP. A pressure transducer converted the IOP into an appropriate input signal for a customized MATLAB routine and allowed to automatize the inflation process. The MATLAB program also controlled the pumping, which changed the IOP by varying the height of the water column. Temperature and humidity within the eye chamber were continuously monitored and recorded
[31]	Corneal Samples: 24 P	Loading: The control tap was carefully operated allowing the air gradually to inflate the cornea while reaching the maximum testing pressure (about 0.8 bar)	The main components of the equipment were an inductive displacement transducer loose plunger and full scale of ± 50 mm; a strain gage pressure transducer with a full scale of 10 bar; an acquisition board NI SCXI-1000 connected to a PC by means of a NI SCXI-1200 USB module. Specifically, the acquisition board was composed of a conditioning module for strain gages NI SCXI-1121 with terminal block NI SCXI-1321 and a conditioning module for LVDT sensors NI SCXI-1540 with terminal block NI SCXI-1315. The compressed air circuit consisted of an air compressor with pressure tank, a tap serving as control valve to regulate air pressure in the test circuit, a T junction connecting the air circuit with the pressure transducer, and a pressure relief tap. All of these components were linked to the cornea holder by means of plastic piping and quick release couplings
[55]	Corneal Samples: 5 P	Loading-Unloading: IOP was changed from 10 to 90 mmHg to inflate and deflate the cornea in one typical test. To simulate the conditions of quasi-static and dynamic loading, two different loading rates of 3.3 and 33 mmHg min^−1^ for IOP change were used to test the cornea	The corneal sample was sealed on its rim on the outer side of an artificial anterior chamber. Within the seal it was filled with normal saline solution, mimicking the aqueous humor in the anterior chamber of human eye exerting an IOP on the cornea. A motor driven pumping system was designed to control IOP by adjusting the height of the saline solution. IOP was quantified by a pressure sensor installed on the sidewall of the water tube

*H* human; *P* porcine; *CXL* Cross-linking; *IOP* IntraOcular Pressure

**Table 3:** Other e*x vivo* mechanical testing

Study	Test	Samples	Test protocol	Setup	Results
[34]	Indentation in creep condition	No. of samples: 11 H control and 11 H CXL-treated triangular specimens	The maximum nominal indentation load was 50 µN. A trapezoidal load-time profile with 30 s loading, 180 s hold period and 30 s unloading was used. These conditions yielded contact radius of ~ 120 μm for indentation depths of ~ 30 μm. At least three measurements in each region were performed with 200 μm distance between individual indents	Corneas were kept in culture medium containing 6% Dextran at 34° C. The epithelium was abraded and the stroma saturated with 0.1% Riboflavin eye drops for 30 min. Controls were continuously exposed to Riboflavin drops for another 30 min while other corneas were irradiated with UVA light (wavelength 370 nm) while continuously being exposed to dripping Riboflavin for another 30 min (Dresden protocol). Samples were sectioned in the form of a triangular segment and completely immersed in culture medium during the indentation with the posterior surface glued to the base of a Petri dish using high viscosity instant glue. The indentation measurements were performed on the anterior surface in the central region (0–1 mm off-center), paracentral region (from 1 mm off-center to 2.5 mm) and peripheral region (from 2.5 mm off-center to 4 mm), with a 500 μm radius indenter made of ruby using a bioindenter	For control corneas, Young’s Modulus 23.2 ± 5.7 kPa, 36.4 ± 12.5 kPa and 43.2 ± 12 kPa respectively in peripheral, paracentral and central regions. For CXL-treated corneas, Young’s Modulus 37.7 ± 20.4 kPa, 65.0 ± 17.9 kPa and 89.9 ± 42.4 kPa respectively in peripheral, paracentral and central regions. Creep value (based on ISO 14,577) 62.3 ± 15.7%, 73.3 ± 30.4% and 61.6 ± 15.0% respectively in peripheral, paracentral and central regions for control corneas, while 52.3 ± 13.2%, 49.1 ± 14.9% and 43.6 ± 14.7% respectively in peripheral, paracentral and central regions for cxl-treated corneas
[47]	Indentation	No. of samples: 15 P eyes CCT: 1.08 ± 0.14 mm	3 cycles of loading and unloading between 5 and 50 mmHg were applied to condition the tissue before testing. IOP was set between 13 and 40 mmHg by adjusting the bottle height. Corneas were indented to a depth of 1 mm at indentation rates of 5, 10, 20, 30, 40 and 50 mm⁄ min after a minimum of 10 min of stabilization	A 10 N load cell (MTS 100-090-795, S-Beam type, load resolution 0.0001 N) was screw-mounted onto the crosshead of a universal testing machine UTM (Alliance RT⁄ 5; MTS Corporation, Eden Prairie, MN, USA). A 5-mm-diameter cylindrical indenter was screw-mounted onto the bottom of the load cell. Eyes were mounted on the test jig and placed under the indenter. The anterior chamber was cannulated and filled with saline via a needle connected to a manometer	Fully contact between indenter and cornea reached for displacement > 0.5 mm. Rate dependence of elastic moduli, calculated from the full contact region on the load-displacement curves, decreased with increasing indentation rate. Elastic moduli resulted rate-independent and elastic at rates above > 20 mm⁄ min, dependent on IOP. Elastic modulus resulted 0.05–0.55 MPa for IOP of 10–40 mmHg
[49]	Stress-relaxation unconfined compression	No. of samples: 8 P circular buttons Diameter: 10 mm Initial thickness: 1293 ± 104 μm	A compressive tare stress of 1.5 kPa was applied to ensure a uniform contact between the sample and loading platens and determine the initial thickness. Samples were compressed with a constant displacement rate of 0.15 μm s^−1^ from their initial thickness to 20% engineering strain in five equal 4% strain increments	The epithelium and endothelium were scraped off. Samples were wrapped in cling films and stored in the freezer at − 20 °C. Prior to testing, specimens were thawed at room temperature and were allowed to equilibrate in 0.9% NaCl solution for an hour. Then, they were placed in the submersion chamber. A strain-controlled rheometer (DHR − 2TAInstruments, Delaware) was used for tests	In-plane Young’s Modulus (average 1.33 ± 0.51 MPa) linearly increased with increasing strain. Out-of-plane Young’s Modulus (average 5.61 ± 2.27 kPa) independent of the compressive strain. Permeability coefficient (average 2.14 ± 0.68 × 10^−14^ m^4^/N s) decayed exponentially with increasing strain
[50]	Stress-relaxation unconfined compression	No. of samples: 15 P circular buttons Diameter: 10 mm Initial thickness: 1276 ± 102 μm	A compressive tare stress of 1.5 kPa was applied to ensure a uniform contact between the sample and loading platens and determine the initial thickness. Samples were compressed with different displacement rates (0.15, 0.50 and 1.00 μm s^−1^). For each step of the five successive stepwise stress-relaxation tests, a 4% constant engineering strain	Samples were wrapped in cling films and stored in the freezer at − 20 °C. Tests were conducted using a strain-controlled rheometer (DHR-2 TA Instruments, Delaware). The rheometer’s software, Trios, was used to record the data and to control the force and displacement (with mN and µm accuracies, respectively). Prior to each test, specimens were thawed at room temperature and allowed to equilibrate in a bathing solution. All tests were performed in 0.9% NaCl solution and at a constant temperature of 37 °C	In-plane Young’s Modulus increased with increasing strain: 0.7 ± 0.2 MPa and 1.6 ± 0.2 MPa respectively for 4 and 16% strain at 0.15 μm s^−1^, while 0.8 ± 0.2 MPa and 2.9 ± 0.6 MPa respectively for 4 and 16% strain at 1.00 μm s^−1^.Permeability coefficient decayed with increasing compressive strain: 3.1 ± 0.6 × 10^−14^ and 1.7 ± 0.3 × 10^−14^ m^4^/N s respectively for 4 and 16% strain at 0.15 μm s^−1^, while 5.4 ± 1.3 × 10^−14^ and 2.6 ± 0.1 × 10^−14^ m^4^/N s respectively for 4 and 16% strain at 0.15 μm s^−1^. By varying loading rates and compressive strains, out-of-plane Young’s Modulus ranged from 0.6 kPa to 13.8 kPa, in-plane Young’s Modulus from 0.5 MPa to 4.8 mPa, and permeability coefficient from 1 to 7 × 10^−14^ m^4^/N s
[52]	Torsional shear at different compressive strain levels	No. of samples: 16 P buttons Diameter: 8 mm	An initial constant axial load of 0.17 N was applied to ensure firm clamping of specimens and experiments at relevant levels of swelling pressure. Dynamics shear tests were performed at 0, 10, 20 and 30% compressive strains. For each strain step, an axial displacement rate of 1 μm s^−1^ and a relaxation time of about 30 min were used. At each level of strain, two types of oscillatory tests were performed: strain sweep experiments were done at frequency 1 Hz over shear strain amplitudes ranging from 0.01 to 10%; frequency sweep experiments were done with frequencies of 0.01–2 Hz and a shear strain magnitude of 0.2%	The epithelium and endothelium were scraped off. A DHR-2 Rheometer (TAInstruments, Delaware) with a minimum torque oscillation of 2 nNm, torque resolution of 0.1 nNm,and displacement resolution of 10nrad was used to perform the experiments. Sandpapers were glued to the loading platens to increase friction and prevent possible slippage. Prior to testing, the specimens were equilibrated in OBSS solution (ALCON laboratories, Inc., FortWorth) for 30 min. The submersion chamber of the rheometer was filled with OBSS solution	Increased shear moduli by increasing the compressive strain. Average shear storage modulus ranged from 2 to 8 kPa and average loss modulus ranged from 0.3 to 1.2 kPa. By varying the shear strain level at the same frequency, loss modulus almost constant, while storage modulus decreased from shear strain larger than about 1.5%

*H* human; *P* porcine; *CXL* Cross-linking; *CCT* Central Corneal Thickness; *IOP* IntraOcular Pressure

Most of studies tested corneas in the healthy condition as they were extracted, while some works investigated also the tissue mechanical behaviour in lesioned and treated conditions. Lesioned corneas with structural damage of the stromal layer, simulating melting ulcers, were obtained by means of a chemical burn due to Alkali solution by soaking the sample in NaOH [24] or by applying on its surface a paper disk soaked with NaOH [14] (Table 1). Moreover, the effect of the CXL treatment on corneal biomechanics was studied by using drops of riboflavin to the sample surface and then UVA irradiation with specific irradiance wavelength, exposition time and distance from the cornea [14, 29–34] (Tables 1, 2 and 3).

The thickness was a crucial parameter, for the quantification of the transverse section in tensile tests, but also for the development of geometries in computational modelling. The thickness was obtained by surgery data [10, 21, 22, 46] or measured by pachymetry [11, 13, 20, 25, 26, 29, 30], digital calliper [14, 24], ultrasonic thickness measurement [32], optical-magnifying scale [12] or microscope [23]. All these methods have some limitations in terms of accuracy due to thickness variation between central and peripherical regions and to tissue swelling during all the experimental procedures for sample preservation and preparation [5, 23]. Only in two studies [5, 47], the exact thickness was acquired during the testing with an optical coherence tomography (OCT) imaging. In general, the measured thickness ranged from 543.96 to 720 μm for human corneas [5, 22, 27, 46] and from 0.80 to 1.76 mm for porcine corneas [11, 20, 24–26, 29]. Before testing, corneas were preserved in Eusol C (Alchimia, Padova, Italy) [5, 10, 11, 20, 21, 46] or Optisol [28, 46] medium or soaked in physiological saline solution (PSS) [14, 25], 0.9% NaCl solution [48–50], phosphate buffer saline (PBS) [17], 5% [19], 6% [34, 51], 15% [27] or 20% Dextran solution [26] or OBSS solution (ALCON laboratories, Inc., FortWorth) [52]. In addition, different methods were adopted to hydrate corneas during the tests: by immersing the specimen in a chamber filled with Eusol-C [13, 15, 20], 5% Dextran [19], NaCl [44, 49, 50] or OBSS solution [52], by dropping PSS on the specimen surface [14], by spraying the sample with PBS [17], by using an ultrasound moistener [26, 27]. To minimize hydration before and during the test, in one study samples were coated with mineral oil [23].

The storage and hydration seemed to affect the mechanical properties of the cornea [15, 28], in particular the stiffness decreased for more hydrated corneas and the mean corneal thickness varied strongly among the different hydration conditions.

#### Uniaxial tensile testing

All corneas were cut in rectangular strips with length ranging from 7 to 20 mm and width ranging from 2.5 to 5 mm [11–17, 19, 20, 22–26, 29, 30], except stromal lenticules (approximately length 6.5 mm and 1 mm width) [5, 10, 21, 46]. Corneal strips were extracted along the superior-inferior (SI), nasal-temporal (NT) and/or diagonal (45° or 135°) anatomical directions.

Most of studies analysed the tensile behaviour only in one anatomical direction of the cornea (SI [13, 17, 20, 23, 29, 30], NT [14, 24]), others in both directions (SI and NT directions [10, 15, 21, 25, 44] or also in diagonal directions [11, 19, 26]) while in the remaining studies the direction was not specified [5, 12, 16, 22, 46].

Regarding the tensile instrument, the majority of the tests were performed by using Instron testing machine (model 3366 [11, 13, 20, 25], 5566 [22], 5544 [17] or 5848 [26]), while others by means of IBTC-50 tensile machine [10, 21, 44, 46], Minimat tester [29, 30], custom-built testing system [5, 24], Bose ElectroForce® Planar Biaxial Test Bench instrument [14], Ustretch device [19] and MTS SynergieEden Prairie [15]. Samples were clamped by machine grips and almost all used the sclera tissue at the ends of the specimen for the mechanical attachment [11, 13–17, 20, 22–26, 30, 44]. Multiple techniques were adopted to avoid the slippage, by using clamps with rough surfaces [10, 11, 20, 21] or with 320-grit sandpaper [26], custom grips coated with water-proof sand paper [17], by cyanoacrylate glue on surface of the ends [23] or by interposing the ends by patches of balsa wood and Velcro [14] or biological material holder [16]. Instead of clamps as other studies, only one study used BioRake (CellScale, Waterloo, Canada) attachments to test thin stromal layers, avoiding the sample squeezing and allowing some lateral contraction of the specimen [19].

**Fig. 1:**
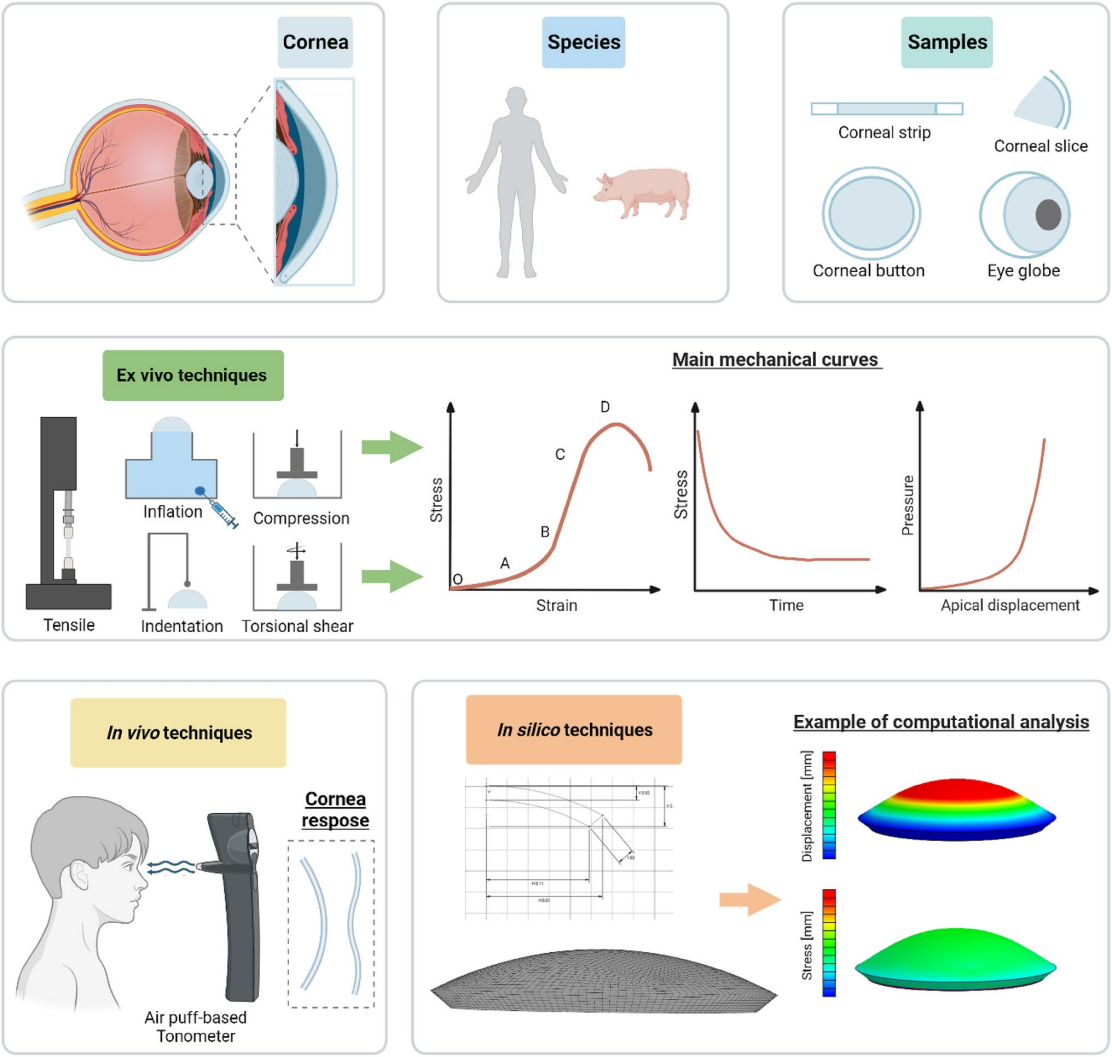
Schematic illustration comparing the ex vivo, in vivo and *in silico* techniques for the mechanical characterization of the cornea. Created with BioRender.com

A greater proportion of studies investigated the short-term mechanical response by stretching the cornea [5, 29, 30, 46] up to failure [10, 11, 15–17, 21–26] or through loading/unloading cycles [19, 20], while few studies investigated the long-term behaviour by stress-relaxation tests [12–17]. In tensile experiments up to failure, the typical stress-strain curve reported a nonlinear behaviour composed by an initial toe region followed by a region of higher stiffness [10, 15–17, 20, 21, 23, 26]. In detail, the curve can be divided in approximately four segments (Fig. 1): at the linear elastic OA segment, stress changes slowly and the strain rises rapidly; at the AB segment, a nonlinear relationship increases exponentially; at the BC segment, the relationship is approximated to a straight line; at the final CD segment, the curve reflected a nonlinear relationship where D is fracture point [10, 21, 26]. The initial two segments generally refer to normal physiological stress state of the corneal tissue (strain less than 0.1) [16, 26], and in particular, in the BC tract the Young’s Modulus is usually calculated as the curve’s slope. On the other hand, in stress-relaxation tests, the characteristic stress-time curve is shown in Fig. 1 and it can be described by an initial peak stress corresponding to the application of the sudden deformation and its subsequent gradual decrease over time up to a steady-state region where all the time-dependent phenomena are completely occurred [14–17]. Moreover, in literature, two additional tensile tests are reported: the contraction test, to measure the transversal contraction index by using an optical method [15], and the trouser tear test considering strips with a sharp notch prior to test (Table 1), to investigate the fracture toughness of the cornea [17]. In trouser tear tests, a quasi-linear increase of the load resulted at small extension up to a plateau region in which the crack started to propagate [17]. The main corneal mechanical properties obtained by the reviewed studies were grouped in terms of Young’s Modulus, failure parameters, stress-relaxation parameters, tear toughness and transversal contraction index, expect those reported by [22, 23]. Mahdian et al. [22] determined the nonlinear anisotropic fiber-reinforced hyper-viscoelastic (AFHVE) model parameters by combining the results of tensile tests up to failure on human corneal strips (Table 1) to the coupled finite element-optimization analysis to predict the tissue behavior. 8 parameters of the AFHVE model were obtained (3 for the viscoelasticity and 5 for the anisotropic hyperelasticity) and their sensitivity was evaluated by studying the convergence of the tissue response between simulation and experiment [22]. Elsheikh et al. [23] proposed a mathematical procedure to remedy the overestimation of corneal stiffness due to the inaccuracy sources of tensile tests, reducing the difference between the stiffness determined by extensometry and inflation tests approximately from 32 to 5%.

##### Young’s modulus

For human stromal lenticules, contrasting results in terms of Young’s Modulus in the early region of the stress-strain curve resulted under the same elongation rate of 0.01 mm s^−1^: for strain less than 5%, [10] obtained slightly higher values in NT direction if compared to the SI (NT: 1.30 ± 0.51 MPa; SI: 1.14 ± 0.28 MPa) while [21] showed higher values in the SI direction at stress of 0.01 MPa (NT: 1.17 ± 0.50 MPa; SI. 1.28 ± 0.70 MPa), 0.02 MPa (NT: 1.46 ± 0.47; SI: 1.57 ± 0.67 MPa) and 0.03 MPa (NT: 1.75 ± 0.45 MPa; SI: 1.84 ± 0.64 MPa).

Similarly, the SI human corneal orientation resulted statistically significantly higher also by considering the low-strain tangent modulus (LSTM) for strain less than 20% (NT: 1.17 ± 0.43 MPa; SI: 1.32 ± 0.50 MPa) and the high-strain tangent modulus (HSTM) for strain between 35% and 55% (NT: 43.59 ± 7.96 MPa; SI: 51.26 ± 8.23 MPa) [21]. By studies on human cornea with no indication about the anatomical direction investigated [5, 46], overall higher values of Young’s Modulus at stress of 0.02 MPa (2.45 ± 1.72 MPa [46]) and lower values of both LSTM (0.204 ± 0.189 MPa) and HSTM (5.114 ± 1.958 MPa) [5] were obtained if compared respectively to the corresponding values reported above [21], when different testing machine was used [5, 46] and the sample thickness was acquired during the testing by OCT imaging [5] instead of the estimation from surgery data. However, the Young’s Modulus reported by [46] resulted negatively correlated to corneal densitometry values in some corneal layers and statistically significantly positively correlated to age, probably due to the mechanical improvement for thicker collagen fibres caused by aging. LSTM and HSTM reported by [5] resulted significantly correlated with a newly introduced in vivo stiffness metric and individually the LSTM resulted positively correlated with the in vivo Corvis ST indicators related to the segment/region of the corneal surface due to the first applanation and the HSTM resulted to decrease with the degree of myopia, in agreement with previous in vivo studies [5].

For full-thickness corneas, generally, porcine samples exhibited an average slope of 3.193 ± 1.589 MPa in the low strain range up to 4% and of 41.806 ± 10.920 MPa for strain range 6 − 12% [15]. In SI direction, Young’s Modulus at 6% strain resulted, on average, 1.5 MPa and 2.7 MPa respectively in untreated and CXL treated porcine corneas and 1.3 MPa and 5.9 MPa respectively in untreated and CXL treated human ones [30]. In addition, along the depth of the tissue, in SI corneal specimens the human anterior layer resulted stiffer than the posterior one (at 5% strain, mean 3.6 MPa and 1.3 MPa for anterior and posterior samples) and a stronger effect of the CXL treatment resulted in the anterior layer (at 5% strain, mean 6.0 MPa and 1.0 MPa for anterior and posterior treated samples) [29]. The same CXL effect resulted for porcine SI corneal specimens (at 5% strain, mean 2.9 MPa [26, 29] and 6.3 MPa for untreated and treated anterior samples, while 2.8 MPa and 2.7 MPa respectively for posterior samples [29]). Therefore, considering both the total thickness and the distinction in layers, a greater increase in stiffness due to CXL treatment resulted in human corneas than in porcine ones [29, 30]. Furthermore, for porcine samples in SI direction, when two corneal layers were distinct from the anterior to the posterior surface, no difference in stiffness resulted from each other (at 5% strain, mean 2.9 MPa and 2.8 MPa respectively for anterior and posterior samples [29]), while, in case of splitting in three layers, the Young’s Modulus was found to decrease gradually along the depth (at the stress of 0.03 MPa, 2.869 ± 0.584 MPa, 2.333 ± 0.337 MPa and 1.640 ± 0.331 MPa respectively for anterior, central and posterior layers) [26]. Similarly, the stiffness reduction along the corneal thickness was also observed in the diagonal direction (at stress of 0.03 MPa 2.706 ± 0.707 MPa, 2.071 ± 0.584 MPa and 1.415 ± 0.228 MPa respectively for anterior, central and posterior layers) [26] and in the NT direction (at stress of 0.03 MPa, 2.484 ± 0.740 MPa, 2.098 ± 0.536 MPa and 1.746 ± 0.386 MPa respectively for anterior, central and posterior layers) [26]. Additionally, the Young’s Modulus for porcine NT specimens was also investigated in control and NaOH-treated conditions respectively about 10 MPa and 5 MPa, showing a significant difference between them [24].

For porcine corneas, the Young’s Moduli in different orientations were proved to be equal to each other in the anterior layer (at stress of 0.03 MPa, 2.869 ± 0.584 MPa, 2.484 ± 0.740 MPa and 2.706 ± 0.707 MPa respectively in SI, NT and diagonal directions) [26], as well as in the central layer (at stress of 0.03 MPa, 2.333 ± 0.337 MPa, 2.098 ± 0.536 MPa and 2.071 ± 0.584 MPa respectively in SI, NT and diagonal directions) [26] and in the posterior layer (at stress of 0.03 MPa, 1.640 ± 0.331 MPa, 1.746 ± 0.386 MPa and 1.415 ± 0.228 MPa respectively in SI, NT and diagonal directions) [26]. Focusing on corneal stroma at depth of 100, 350 and 600 μm, lower stiffness in the most posterior layer was confirmed even in this case [19], as well as the independence from the orientation for the anterior layers, contrary to the most posterior one exhibiting a stiffer response in the SI direction compared to the NT and diagonal directions [19]. Such stiffer behavior in SI orientation was reported also by [15], with SI porcine specimens 21% more rigid than NT ones, with significant differences for strain levels higher than 12%.

In porcine corneas, the Young’s Modulus was also studied for different strain rates [11, 17, 20] and results are related to the protocol adopted. By testing the same porcine specimen under different strain rates ranging between 0.8 and 430% min^−1^ (Table 1), a large (40.2% on average) and statistically significant increase of stiffness in SI orientation resulted only from 0.8 to 8% min^–1^, while subsequent increases in strain rate led to much lower and not statistically increase in stiffness [20]. Moreover, when different porcine specimens under different strain rates were compared, the Young’s Modulus in SI direction showed the insensitivity to the rate not only considering high and distant values (9.59 ± 1.28 MPa, 10.29 ± 0.85 MPa and 9.82 ± 1.39 MPa respectively for 3, 30 and 300 mm min^−1^ [17]; for 0.01 MPa stress, 0.349 ± 0.053 MPa and 0.397 ± 0.056 MPa respectively for 8.3 and 210% min^−1^ [11]; for 0.0 MPa stress, 1.036 ± 0.155 MPa and 1.181 ± 0.164 MPa respectively for 8.3 and 210% min^−1^ [11]), but also fr low strain values (for 0.01 MPa, 0.343 ± 0.064 and 0.349 ± 0.053 MPa respectively for 0.8 and 8.3% min^−1^ [11]; for 0.03 MPa stess, 1.016 ± 0.183 MPa and 1.036 ± 0.155 MPa respectively for 0.8 and 8.3% min^−1^ [11]). Similarly, small icreases of the stiffness by increasing the strain rate from low to high values were obtained also in the other orientations in porcine corneas, the NT direction (for 0.01 MPa stress, 0.379 ± 0.054 MPa, 0.382 ± 0.030 MPa and 0.423 ± 0.031 MPa respectively for 0.8, 8.3 and 210% min^−1^, for 0.03 MPa stress, 1.14 ± 0.155 MPa, 1.133 ± 0.090 MPa and 1.252 ± 0.090 MPa respectively for 0.8, 8.3 and 210% min^−1^) and the diagonal directin (for 0.01 MPa stress, 0.374 ± 0.022 MPa, 0.426 ± 0.103 MPa and 0.427 ± 0.037 MPa respectively for 0.8, 8.3 and 210% min^−1^; for 0.03 MPa stress, 1.19 ± 0.065 MPa, 1.249 ± 0.302 MPa and 1.264 ± 0.111 MPa respectively for 0.8, 8.3 and 210% min^−1^) [11]. In addition, for 0.8, 0.3 and 210% min^−1^ strain rates, SI and NT speciens had almost the same stiffness and appeared to be slightly stiffer than diagonal ones by about 2–13%, except for the 0.8% min^−1^ for which diagonal samples resulted stiffer than the NT ones by 12% [11], while for values from 0.1 to 50 mm min^−1^ the SI porcine specimens resulted on average 34% stiffer than the NT ones [25].

##### Failure parameters

The tensile behaviour up to failure can be described by means of the tensile strength, or named failure stress, as the maximum stress that the tissue can sustain before failure, the yield stress/strain, as the stress/strain at which a permanent deformation begins, and the failure strain, as the strain at which the failure happens.

The tensile strength resulted slightly larger in SI direction rather than NT direction for both human stromal lenticules (SI: 14.05 ± 1.95 MPa; NT: 13.25 ± 2.16 MPa) [10] and porcine corneas [25]. Under the same strain rate of 10 mm min^−1^, the average tensile strength resulted very similar for human (3.81 ± 0.40 MPa) and porcine corneas (3.70 ± 0.24 MPa), as well as the stress-strain pattern [12]. Lower values of porcine tensile strength were obtained when only the NT direction was investigated, both in control and NaOH-treated conditions (respectively about 2.5 MPa and 1 MPa) [24].

Additionally, the porcine corneal behaviour up to failure was also described, on average, by yield stress of 3.837 ± 1.312 MPa, yield strain 15.4 ± 2.4%, failure stress 4.763 ± 1.251 MPa and failure strain 19.2 ± 2.3% [15], with higher yield stress and higher failure stress in SI specimens with respect to NT ones [15].

##### Stress relaxation parameters

Generally, the stress-relaxation behaviour can be described by the peak stress, the stress at the end of relaxation, the relaxation percentage and the time constant [15]. Porcine corneas for 2% strain constant for 50 s exhibited a relaxation stress percentage of 16.1% from a peak stress of 0.059 ± 0.039 MPa with a time constant of 6.165 ± 1.649 s, while for 10% strain constant for 2000 s respectively the corresponding values 0.322 ± 0.049%, 0.654 ± 0.611 MPa and 471.101 ± 75.229 s [15]. The strain-dependence of the stress relaxation was observed also in [17], where the equilibrium normalized load resulted in 0.19 ± 0.02 and 0.41 ± 0.02 respectively for 2 and 4 mm displacements fixed for 120 s. On the other hand, considering stretch level up to 4 mm applied for 1000 s [16], porcine corneas exhibited, on average, the initial and the last stress respectively as 2.33 MPa and 0.88 MPa and the stress-relaxation curve was fitted by the modified Maxwell viscoelastic model (relaxation modulus E_1_: 0.69 MPa, E_2_: 0.43 MPa, E_3_: 0.31 MPa, E_4_: 0.40 MPa, E_5_: 0.43 MPa; relaxation time τ_1_: 8.83 s, τ_2_: 65.33 s, τ_3_: 876.93 s and τ_4_: 2.84 × 10^3^ s) [16].

When human corneal strips of 10-mm initial length were stretched and held for 400 s with the length reached under the 4 N load [13] (Table 1), the average stress reductions resulted in 27.7 ± 5.6%, while 30.5 ± 5.7% after 800 s and 32.0 ± 5.7% after 1200 s respectively with the length under the 8 and 12 N loads [13]. The corresponding average stress reductions for porcine corneas was 49.2 ± 8.3%, 55.6 ± 8.2% and 5.2 ± 8.1% respetively after 400, 800 and 1200 s [13]. In addition, the stress-relaxation behaviour can be described also by the slope *K* of the normalized relaxation *G(t)* curve in function of a log time *t* and the value *P* of *G(t)* at the end of relaxation. For a stretch ratio of 1.5 (Table 1), the slope K of the G(t)-ln t and the value P for relaxation of 1000 s resulted respectively 0.0165 ± 0.0024 and 85.6 ± 1.5 for human corneas, while 0.0553 ± 0.0069 and 64.6 ± 3.3 corresponding values for porcine corneas [12]. Therefore, these studies [12, 13] showed that porcine corneas relax quicker and lose more of their initial stresses than human ones with statistically significant differences in relaxation rates [12, 13].

Moreover, stress-relaxation tests on porcine corneas were performed also in alkali-lesioned and CXL-treated conditions and following preservation in culture medium [14] (Table 1). The time-dependent behaviour of the porcine cornea resulted in being influenced by the structural modifications due to both the lesion and the CXL treatment, showing lower relaxation times if compared to the healthy corneas both in fresh and cultured conditions [14].

##### Tear toughness

Tear toughness of the cornea was calculated at different extension rates considering the peak force, defined as the mean load at the plateau region of the load-extension curve and the thickness of the specimen [17]. Peak force resulted larger for greater extension rates and the tear toughness exhibited values from 3.39 ± 0.57 to 5.40 ± 0.48 kJm^−2^, depending on the extension rate [17]. A linearly positive relationship resulted between the tear toughness and the extension rate in a semi-log plot and toughness increasing approximately by 1.00 kJm^−2^ for everyone order of magnitude increase in extension rate [17].

##### Transversal contraction index

The transversal contraction index was measured in porcine corneas which exhibited in general values greater than 0.5, confirming the tissue nonlinearity and anisotropy, and dependent on strain level: 1.005 ± 0.496, 2.082 ± 0.196 and 1.908 ± 0.237 respectively for strain of 5, 10 and 15% [15].

#### Inflation testing

Inflation testing has taken hold with the aim of keeping the tissue intact loading the whole structure with an internal pressure that simulates intraocular pressure. The pressure is applied by fluid injection using a water column or syringe pump that can be controlled by computer [53]. In inflation tests, the deformation of the specimens that result from the change in internal pressure is measured by a system of high-resolution digital cameras that are spatially distributed around the specimen and, together with the initial specimen size and applied pressure, is analyzed using shell analysis to determine the stress-strain behaviour of the tissue. In contrast to uniaxial tensile tests, the inflation test does not involve flattening of the tissue and separation of collagen fibrils along the edges of the specimen and for these reasons, preconditioning is rarely performed [54].

Samples may consist of the corneal component alone or the entire eyeball. Corneas are mounted on a pressure chamber and held in place using mechanical clamps. For whole globes either a needle connected to the pumping system is introduced through the optic nerve head or an air puff acting from the outside hits the cornea directly. Different protocols were used to perform inflation tests, details are reported in Table 2. The main tests consisted of cycling, creep and stress relaxation tests.

The relationship between intraocular pressure and apical elevation of the cornea was analysed in several studies [13, 31, 32, 55] on both porcine and human corneas, even in the presence of treatment, and the results showed the non-linearity of the mechanical response. The initial behaviour or toe region is located in different pressure ranges: up to 10 mmHg [13], in 12–15 mmHg range [32] or in the 2-4 kPa as physiological range [31]. Human corneas showed low stiffness up to pressure values of about 15–20 mmHg, while later stiffness increased [13]. Furthermore, human eye showed higher stiffness at the limbal region reducing gradually towards the posterior pole, while the central cornea had a higher stiffness than the peripheral cornea [51]. Pig corneas showed nonlinear behaviour with a less gradual transition respect human ones from the initial stage of low rigidity to the final stage with greater rigidity. Porcine corneas treated with CXL had a lower corneal apex elevation at all loading stages compared to untreated corneas [31]. Moreover, the relationship between Young’s Modulus (calculated on the basis of shell theory [56]) and IOP appeared to be linear. Bao et al. [32] demonstrated that porcine corneas treated with CXL had a higher stiffness (Young Modulus 356 ± 159 kPa) respect untreated ones (Young Modulus 247 ± 169 kPa) in the region 2–4 kPa. The average Young’s Modulus of the human anterior cornea ranged between 2.28 and 3.30 MPa in specimens with and without intact epithelium, respectively, while for the posterior cornea was on average 0.21 and 0.17 MPa, respectively [27].

The stress-strain relationship was also identified using the pressure rise and the specimen dimensions, resulting non-linear as well. In details, the study by Elsheikh et al. [13] for human corneas reported an exponential equation that was derived for each age range, while a third-grade polynomial equation for the porcine corneas. A comparison among species revealed that porcine corneas appeared to have lower initial and final stiffness values than all groups of human corneas. In the creep tests [13], a gradual decrease in mean human corneal creep was recorded with the increase of age. In addition, porcine corneas experienced significantly more creep deformation than all human corneas, and the overlap between porcine and human creep time results was limited to the early stages of the test, beyond which there was a clear separation. Also, another study [27] reported differences between anterior and posterior human cornea with and without epithelium. The results showed that the anterior cornea of specimens with epithelium took a longer time to relax compared to that without epithelium and when the load was released the specimens with epithelium recovered corneal strain faster. The posterior cornea of specimens with epithelium showed a slower creep response to sudden stress application than specimens without epithelium. After sudden stress release, no immediate elastic recovery of the posterior cornea was found in a few samples. A permanent strain was found immediately after stress release [27]. In Kling et al. [33], corneal buttons and whole eye globes were compared showing that the sclera slightly affected the temporal symmetry, while the ocular muscles drastically changed the amount of corneal recovery. CXL produced a change in the viscoelastic properties with treatment. In another study, Kling et al. [28] demonstrated that the mechanical response was influenced if the corneas were treated with different solutions. Dehydration induced by dextran solution increased the hysteresis after the inflation cycle, while corneas treated with 0.125% riboflavin–20% dextran recorded higher strains, indicating a softening of the corneal tissue compared to untreated corneas and corneas treated with 8% dextran and 20% dextran.

#### Other mechanical testing

Other ex vivo methods were also used to study the mechanical properties of cornea (Table 3). By indentation test in creep condition, the effect of the CXL treatment was studied in human corneas (age ranged from 48 to 98 years) [34]. A significant increase in Young’s Modulus from the peripheral to the central region resulted for both the control (23.2 ± 5.7 kPa, 36.4 ± 12.5 kPa and 43.2 ± 12 kPa respectively for peripheral, paracentral and central regions) and CXL-treated corneas (37.7 ± 20.4 kPa, 65.0 ± 17.9 kPa and 89.9 ± 42.4 kPa respectively for peripheral, paracentral and central regions) [34]. In addition, the stiffness of the central and paracentral regions increased almost two times after the crosslinking, while not significantly difference resulted for the peripheral region. The creep value, defined according to ISO 14,577, resulted lower in the central and paracentral regions for both the control (62.3 ± 15.7%, 73.3 ± 30.4% and 61.6 ± 15.0% respectively for peripheral, paracentral and central regions) and the CXL-treated corneas (52.3 ± 13.2%, 49.1 ± 14.9% and 4.6 ± 14.7% respetively for peripheral, paracentral and central regions), with a ~ 30% lower value for the treated samples in the central and paracentral regions rather than in control corneas for the same regions [34].

Ex vivo indentation was performed also on porcine corneas to test a new method to measure the IOP-dependent corneal tangent modulus in vivo [47]. Results showed corneal elasticity ranged from 0.05 to 0.55 MPa for IOP from 10 to 40 mmHg and rate-independent at rates > 20 mm min^−1^.

Additionally, two studies performed unconfined compression tests on porcine corneal stroma (Table 3) and determined the out-of-plane Young’s Modulus as the slope of the equilibrium stress-strain curve and the in-plane Young’s Modulus and the permeability coefficient by fitting the experimental data with a transversely isotropic bi-phasic model [49, 50]. By increasing the compressive strain, the in-plane Young’s Modulus (on average, 1.33 ± 0.51 MPa) linearly increased with increasing strain while out-of-plane Young’s Modulus (on average, 5.61 ± 2.27 kPa) was almost independent of the compressive strain [49]. On the other hand, the permeability coefficient (average 2.14 ± 0.68 × 10^−14^ m^4^/N s) decayed exponentially with increasing strain [49].

Considering also different displacements rates, the in-plane Young’s Modulus increased with increasing strain (such as, 0.7 ± 0.2 MPa and 1.6 ± 0.2 MPa respectively for 4 and 16% strain at 0.15 μm s^−1^, while 0.8 ± 0.2 MPa and 2.9 ± 0.6 MPa respectively for 4 and 16% strain at 1.00 μm s^−1^), while the permeability coefficient decayed with increasing compressive strain (such as 3.1 ± 0.6 × 10^−14^ and 1.7 ± 0.3 × 10^−14^ m^4^/N s respectively for 4 and 16% strain at 0.15 μm s^−1^, while 5.4 ± 1.3 × 10^−14^ and 2.6 ± 0.1 × 10^−14^ m^4^/N s respectively for 4 and 16% strain at 0.15 μm s^−1^) [50]. Regardless of loading rates and compressive strains, a range of out-of-plane Young’s Modulus of 0.6 kPa to 13.8 kPa, in-plane Young’s Modulus of 0.5 MPa to 4.8 MPa, and permeability coefficient of 1 to 7 × 10^−14^ m^4^/N s were found [50], in accordance to [49] .

Furthermore, Dynamo-Mechanical Analysis tests were conducted according to torsional shear conditions at different levels of compressive strain on porcine corneas [52], showing average shear storage modulus and loss modulus respectively from 2 to 8 kPa and from 0.3 to 1.2 kPa and increased shear moduli by increasing the compressive strain. By varying the shear strain level at the same frequency, the loss modulus was almost constant, while the storage modulus exhibited a sudden drop at shear strain larger than about 1.5%. In addition, at the same shear amplitude, the storage modulus increased at each compressive strain step [52].

### In vivo studies

In vivo techniques are gaining importance in clinical ophthalmology for the assessment of corneal biomechanics because of the need to avoid the disruptive action performed by ex vivo experimentations. Techniques currently available and in development can be divided between perturbatory, consisting in imposing an external load, and non-perturbatory techniques that collect innate properties of corneal tissue [57]. In the first category, ORA and Corvis ST appear in the in vivo biomechanical testing characterized by high-speed and high-magnitude dynamic deformation of the cornea under air-puff excitation to identify the abnormities in the morphological and biomechanical properties of the cornea. They combine in vivo optical imaging systems with an in-situ non-contact air-puff tonometer, thus they can assess a series of high-speed dynamic biomechanical parameters of the cornea [58–62]. They are the only tests used in clinical medicine. Digital Imaging Correlation (DIC) method was applied to the in vivo high-speed corneal deformation measurement in combination with the Corvis ST tonometer, permitting spatiotemporal dynamic strain/strain rate maps of the cornea at the tissue for the clinical recognition and diagnosis of keratoconus at a more underlying level [63] .

The promising aspect of Corvis ST consists in the possibility to link Corvis ST data to material stiffness parameter, which are quantities more used to describe biological tissues, by using a new algorithm, the stress-strain index (SSI) algorithm, which proposed.

a stress-strain curve for a given cornea based on finite element modelling and generates a property that is largely independent of IOP and corneal thickness, two confounders of biomechanical property measurement [64].

A study with a follow-up of about 41 months revealed a significant reduction in corneal stiffness expressed by a significant reduction in the SSI, demonstrating that in-vivo biomechanical deterioration occurred with keratoconus progression [65].

Other not-used-in-clinics techniques are Brilluoin optical microscopy [66, 67] that, together with Phase-decorrelation OCT [57], corneal indentation [68], estimation of Young’s Modulus based on a fluid-filled spherical shell model with Scheimpflug imaging [69] and ultrasound surface wave elastography [70–72] and optical coherence elastography to measure shear modulus of the human cornea [71] fall into the non-perturbatory techniques. Despite the capability of evaluating in vivo corneal biomechanics in combination with in-situ loading and/or imaging technologies, these methods still face challenges in addressing the issue on high-speed biomechanical measurements and the nonlinear material behaviour of the cornea.

###  In silico studies

As reported in the previous paragraphs, an optimal understanding of corneal biomechanical features is essential to analyse not only the refractive surgical procedures and their consequences in terms of stiffness and microstructure, but also the effects of corneal treatments, such as CXL, in order to predict side effects and avoid them, to detect eventual weakening or enhancement of corneal mechanical response and to improve the management of ectatic corneal diseases. Advances in computational modelling have the potential to enhance diagnosis, enable personalized risk assessment, and optimize treatment design toward the goal of improving safety and outcomes for corneal and refractive surgery patients [73].

At the state of art, FE models of cornea and whole eye have been developed for different purposes: to prevent complications after modern laser refractive surgical procedures and iatrogenic ectasia [74], to study the mechanical response of the cornea subjected to a non-contact air-jet tonometry differentiating the contributions related to geometry, the corneal material behaviour and the loading [75], to analyse the blunt impact of foreign bodies [3], to study the effect of surgical factors on the cornea, to improve keratoconus treatment [41, 76, 77], to analyse the wave propagation for a better understating the differences between healthy subjects and glaucoma patients [78], to measure the deformation after airbag impact [79] and to quantify the biomechanical change caused by the LASIK flap [80]. The most ambitious goal stays on the development of a daily clinical computational tool for the planning and optimization of corrective procedures and in preclinical optimization of diagnostic procedures [40], permitting the personalization of the surgical procedure [81] by using a multi-physics model that permits not only to analyse biomechanical aspects, but also treatments, as CXL, by adding the migration of the riboflavin (i.e., the photo-initializer), UV light absorption, the photochemical reaction that forms the cross-links, and biomechanical changes caused by changes to the microstructure [82].

For what concerns the biomechanical aspects, the geometry and the constitutive formulation of the material vehiculate the outputs. Numerous studies simplified the cornea as a linear, time-independent and isotropic material [76, 78, 79], omitting the “true” mechanical aspects of corneal tissues. More sophisticated papers described the corneas as a material presenting all or part of the following features: anisotropy, visco-hyperelasticity and inhomogeneity [3, 40, 74, 75, 81], respecting more the experimental evidences and permitting more solid simulations-derived considerations.

The ideal geometry consists of a 3D patient-specific cornea (or whole eye ball) [75] obtained from biomedical images or tool as Pentagram or Corvis, avoiding spherical shape, with a thickness differentiated by region. The FE mesh is another crucial parameter, and the number of nodes and elements depends on the geometry, element type and the level of accuracy that has to be reached. A sensibility mesh analysis is encouraged with a proper number of elements describing the thickness. More details about the FE models are reported in Table 4.

**Table 4:** *In silico* studies

Study	Goal	Geometry	FE Discretization	Material Characteristics and formulation	Software	Boundary Conditions
[40]	Development of a daily clinical tool for the planning and optimization of corrective procedures that can be used also in preclinical optimization of diagnostic procedures	Geometry as a semi non-spherical calotte. Thickness values rotationally symmetric; central thickness of 545 μm and peripherical thickness 150 μm greater than the central one	The models consisted of three layers and 24 rings of elements C3D15H containing nine integration points	Hyperelasticity Anisotropy Age-related Viscoelasticity Variation of fibril density/inhomogeneity Near incompressibility of the corneal stroma *Constitutive model*: ad hoc UMAT writing for material formulation	Abaqus	Corneal inflation at 3.75 and 37.5 mmHg min^−1^ and shear test at 10% min^−1^ deformation
[51]	The main objective of this study was to provide a method for determining the material stiffness of the eye	Geometry derived from processing the images obtained from experimental samples	Seventy circumferential rings of quadratic, wedge-shaped, hybrid elements C3D15H	Hyperelasticity *Constitutive model*: First-degree Ogden model	Abaqus	Increasing IOP up to 60 mmHg with a rate of 40 mmHg min^−1^
[74]	Development of a model to prevent complications after modern laser refractive surgical procedures and iatrogenic ectasia. The models could be applied to estimate the difference between an actual IOP and a measured IOP associated with myopia corrections of different D levels	The geometry was defined by the outer corneal radius, 7.4 mm, consisting of the six layers	More than 0.138 million quadratic full-integration mixed-formulation solid elements	Hyperelasticity Anisotropy Near incompressibility *Constitutive model*: Holzapfel model	Comsol Multiphysics	clamped limbus and its surrounding tissues at the edge and liquid pressure on the inner surface equal to 15 mmHg
[75]	The objective is to study the mechanical response of the cornea subjected to a non-contact air-jet tonometry studying separately the contribution of the geometry, the material behavior and the loading because it is not possible with standard non-contact tonometry devices	Patient-specific finite element model of a healthy eye, The corneal topographic map reconstructed using a Pentacam system and the pachymetry data. The model accounted for three different parts: the cornea, the limbus and the sclera	13,425 quadratic full integration mixed formulation solid elements and 62,276 nodes	Hyperelasticity Anisotropy *Constitutive model*: Gasser-Holzapfel-Ogden constitutive equation	Abaqus	Axial displacements and rotations were restrained at the bottom surface of the sclera, three values of IOP (10 mmHg, 19 mmHg and 28 mmHg)
[3]	The goal was to analyze the blunt impact of foreign bodies on the eye to measure the influence of IOP on the amount of force applied to the cornea during tonometry	Asymmetrical 3-D model of the human eye constructed consisting of cornea and sclera under tonometry. The model also incorporates a tonometer cylindrical probe of 1.7 mm diameter	The average element size of the complete eye model is 1.9816 mm. The mesh model resulted in a total node count of 58,999 and a total element count of 11,613	Viscoelasticity Fiber-reinforced for the corneal tissue. *Constitutive model*: Material defined by using experimental nonlinear stress–strain data	ANSYS	The sclera was imposed with displacement zero. The intraocular pressure inside the eye is fixed normal to the sclera and cornea surface from 10 to 20 mmHg
[81]	Development of a methodology to personalize ICRS refractive surgery regarding the patient’s keratoconus stage in order to facilitate the efficiency and biomechanical stability of the surgery	3D patient-specific model of the keratoconic cornea	Linear hexahedral elements of the C3D8R. more than 25,000 elements	Hyperelasticity Anisotropy *Constitutive model*: Modified Gasser-Holzapfel-Ogden	Abaqus	The contact behavior between the intracorneal layer was defined as “frictionless” and “hard” contact. The movement of the layers under physiological IOP was defined by displacement boundary conditions for the upper and lower corneal layer
[76]	Analyzing the effect of surgical factors on the deformation and curvature of the cornea to improve the accuracy of keratoplasty and derive the optimal surgical factors using finite element method	The corneal surfaces were idealized as being smooth and axisymmetric calotte.	8-noded solid element, C3D8, with distortion, hourglass mode control	Isotropy *Constitutive model*: Linear elastic materials	Abaqus	The suturing tension was set to 0.1 N, 0.3 N, and 0.5 N, and the suturing path depth was set to 0.35 mm, 0.45 mm, and 0.55 mm. IOP was set to 15 mmHg
[78]	The goal was to analyze the in vivo data of the wave speed of cornea between healthy subjects and glaucoma patients. This noninvasive method may be useful to measure the in vivo elastic properties of ocular tissues for assessing ocular diseases	Reconstructed from image analysis of the surface profile at a reference pressure of 0 mmHg	Quadrilateral elements (type CPS4R) with size 0.25 mm × 0.25 mm for cornea and 0.5 mm × 0.5 mm for sclera and gel, enhanced with hourglass control and reduced integration	Isotropy Viscoelasticity Nearly incompressibility *Constitutive model*: Linear, viscoelastic generalized Maxwell model	Abaqus	A uniform pressure was applied on the inner surfaces of the cornea and sclera in the direction normal to the corneal and scleral surfaces at each point. The range of IOP was set between 5 and 30 mmHg at an interval of 5 mmHg
[77]	This study was aimed at employing a combination of clinical data, finite element method, and artificial neural network to establish a novel biomechanical-based diagnostic method for the keratoconus eyes	The FE model of each cornea was made on the basis of data provided by the Corvis device	Hexagonal elements. Elements and nodes were 3664 and 5307, respectively	Hyperelasticity *Constitutive model*: 5-parameter Mooney-Rivlin hyperelastic model	Not Reported	The cornea was fixed at two end sides and the two different pressures were applied on that. The pressure as a result of air puff from the exterior side and an intraocular pressure from the interior side of the cornea
[82]	Development of a multi-physics model that considers the migration of the riboflavin (i.e., the photo-initializer), UV light absorption, the photochemical reaction that forms the cross-links, and biomechanical changes caused by changes to the microstructure	Generic biconic surface equation to generate corneal geometry	The entire domain is discretized into 3268 8-node brick elements with 6 elements spanning the thickness	*Biomechanics*: The extracellular matrix is modelled as an incompressible neo-Hookean material. *Radiative transfer*: intensity of the UV light modelled through the thickness of the cornea. The light was considered as monochromatic and the speed of light is significantly larger than the time scale of the other phenomenon. No scattering and emission. *Migration of riboflavin*: migration of the riboflavin is governed by a diffusion isotropic reaction equation. *Photochemical reaction*: polymerization reaction consisting of three phases: (1) initiation, (2) propagation, and (3) termination	Abaqus	Initial conditions: no riboflavin, and a stress-free undeformed body. For the photochemical boundary conditions, riboflavin with a concentration of c0 = 0.1% and a UV light with an intensity of I0 = 3 mW/ cm^2^ Photochemical boundary conditions as a function of time. Symmetry conditions. Both families of collagen fibrils share a dispersion parameter of = 0.1
[41]	Studying the corneal deformation undergoing implantation of realistic and hypothetical ring geometries to assess if intracorneal asymmetric ring segments with varying thickness and base width can be a good alternative in corneas with asymmetric keratoconus phenotypes	Geometry was composed of a three-layered corneal tissue (epithelium, anterior and posterior stroma) fixed at the limbus. The implantation of a triangular-shape asymmetric ring segment with varying ring thickness (150 to 300 μm) and base width (600 to 800 μm) was simulated	A quadrilateral mesh consisting of 385 elements of PLANE183 (8-node structural solid)	Anisotropy *Constitutive model*: transversely isotropic cornea	Ansys	The intraocular pressure was simulated by applying a surface pressure of 15 mmHg to the posterior surface. A radial and circumferential pre-strain of 0.015 in the anterior and 0.010 in the posterior stroma was assigned to account for the deformation induced by applying an IOP of 15 mmHg
[83]	The aim of this study was to numerically simulate a change in the geometry of the corneal coating (refractive surgery) and to verify the equation for correcting GAT readings after refractive surgery	A cornea with a natural geometry where its outer profile and inner profile are described with an ellipse with an eccentricity of 0.5	Because of the assumed symmetry of the eyeball, plane 2D, quadrilateral and 8-node body of revolution elements were used	*Constitutive model*: Material defined by exponential tensile and linear compressive stress-strain curve	Abaqus	Each of the three models was solved after the ablation using the IOP: 16 mmHg, 32 mmHg and 48 mmHg
[79]	FEM for evaluation of the deformation of an intact eyeball of various axial lengths induced by an airbag impact at various impact velocities.	The corneal surface has an aspheric shape, steeper centrally and flatter peripherally. For simplicity, the cornea was assumed to be spherical, with a central thickness of 0.5 mm and a central radius of curvature of 7.8 mm	The cornea and sclera were modelled as membranous elements (shell)	*Constitutive model*: Isotropic linear elasticity	PAMGENERIS	Impact velocities of the airbag patch on the face were 30, 40, 50 and 60 m s^−1^
[80]	The aim of the study was to quantify evaluate the biomechanical change caused by the LASIK flap	The central thickness of the cornea was ~ 0.55 mm. The diameter of the corneal flap was 8.1 mm, and the thickness of the corneal flap was presumed to be 90, 120, 150, 180, 210, and 240 μm, respectively	The size of meshes was selected to be 0.20–0.30 mm. The corneal model comprised 5708 units and 9753 nodes.	Hyperelasticity Nearly incompressible behavior *Constitutive model*: Ogden Hyperelastic Material model (*N* = 1) using experimental stress-versus-strain data.	Not Reported	The corneal boundary and other tissues, such as the sclera, were restrained and firmly fixed by the surrounding biological tissues, including the ciliary processes and iris, as a shell of which the bottom edges are clamped; therefore, the restrained bottom interface of the cornea could be considered to be a clamped boundary
[84]	A methodology to estimate the elastic constants that characterize the constitutive equations that describe the in vivo biomechanical behavior of the human cornea for each patient	Geometry obtained from the processing of images obtained from Corvis	A 2D finite element mesh was constructed from the initial 2D slice using triangular elements. The nodes of the mesh corresponding to the edge of the peripheral cornea were restricted in all directions representing the anchoring with the sclera, which is thought to be 5 times stiffer than the cornea	*Constitutive model*: A second-order hyperelastic Ogden model	MATLAB	The air jet was applied at the apex of the cornea with an average force of 15 mmHg
[85]	Development of an inverse algorithm and 3-D finite element representation of the whole eye for determination of nonlinear, fiber dependent biomechanical properties of the cornea. The biomechanical estimation included the contribution of the cellular matrix, in-plane collagen fibers and cross-links between lamellae that provide the depth-dependent shear resistance	Patient-specific corneal tomography	8-noded hexahedral linear elements for an incompressible material. Four layers of elements were modelled through the corneal thickness	*Constitutive model*: Fiber-dependent hyperelastic model	Abaqus	The distribution of air-pressure on the anterior corneal surface derived from the CFD simulations was applied as a boundary condition normal to the anterior epithelium surface. The pressure is a function of time and radius measured from the geometric center of the cornea
[86]	From Ocular Response Analyzer (ORA) experimental results to develop a method to determine corneal biomechanical parameters	Profile of the cornea obtained from ORA measures	C3D8R mesh	*Constitutive model*: linear viscoelastic material	Abaqus	The quantitative relationship between corneal biomechanical parameters and ORA output parameters is established by combining parametric analysis with finite element simulation

The results proposed by the numerous analysed studies differed in the mechanical quantities that were proposed and varied according to the imposed boundary conditions, the aim of the study and the methodology adopted. For these reasons, the potentialities and the main evidence are reported below but for details the authors invite to refer to the corresponding paper (a list is reported in Table 4).

FE models can analyse vast scenarios and consider the multiple factors, which play a role in the results, differing each single contribution, while this differentiation is not possible in experimentations, unless having at disposal a varied and numerous animal/human samples, making the tests expensive and time-consuming. In fact, Whitford et al. [40] isolated the age-related stiffening behaviour from the age-related viscoelastic changes and Shih et al. [74] found out that the Bowman’s membrane and Descemet’s membrane accounted for 20% of the bending rigidity of the cornea and became the force pair dominating the bending behaviour of the cornea. Also, the relationship among the main quantities as corneal apex displacement, internal ocular pressure (IOP), global and local stress and strain were analysed: Ariza-Gracia et al. [75] showed that the maximum apex displacement varied linearly with IOP, while it followed a cubic relation with corneal thickness. For what concerns the corneal pathology analysis, Karimi et al. [77] revealed the important role of stress among healthy and keratoconus eyes, since healthy eyes showed a higher amount of stress compared to the keratoconus ones, leading to a bigger and more tolerated deformation in health eyes compared to the keratoconus ones. Baek at al [76]. studied the effect of surgical factors on the deformation and curvature of the cornea, finding out that if the suturing depth increased, the local curvature did not significantly change, but the contact position between the thread and the cornea was modified. Curvature variations eventually led to changes in light refraction, which greatly affected the recovery of the patient’s visual acuity. Bagheri et al. [81] and De Oteyza et al. [41] studied the intracorneal ring segment as a keratoconus treatment finding that increasing ring thickness and base width along the arc of the asymmetric ring segment produced a more pronounced flattening in this part of the ring. The asymmetric ring design did find a good balance between maximizing corneal flattening at one end and minimizing it at the other end, compared to the isolated effect of ring thickness and width. Fang et al. [80] studied the LASIK surgery and affirmed that the changes in the biomechanical properties of cornea after refractive surgery should not be ignored because the results showed that the corneal flap can cause the deformation of the anterior cornea and the displacement was enough to change aberrations and stress/strain distribution.

## Discussion

In this work, corneal biomechanics has been studied considering ex vivo, in vivo and *in silico* techniques, highlighting the differences among methodologies, instruments, protocols, and conditions applied, and analysing them by their intended purpose.

All the reported experimental and numerical studies were affected by limitations, some of which inevitable because of the need to simplify the physiological eye conditions, while others more related to the nature of tests.

Comparing different studies result almost impossible for several reasons. A single experimental curve (such as stress-strain relation) has a limited meaning in the comprehensive assessment of the tissue biomechanical properties, as each mechanical test and each specific protocol allow to study only one feature. In addition, there are many variables that can influence the accuracy of the results and cause the discrepancy among studies, as the origin of the samples, the presence of the epithelium and/or endothelium, the methods for preservation and hydration, the testing machine and protocol and the experimental conditions [5, 15] according to the tissue mechanical properties intended to be analysed. Higher values of loading rate (such as, 4.17 mm s^−1^ [12] or 80 mm s^−1^ [14] ) were usually used to simulate an ideally instantaneous application of a certain deformation level in case of stress-relaxation tests, while lower values were considered when the interest laid on the tissue response during elongation (such as, 0.01 mm s^−1^ [10, 21, 46] for tensile up to failure). There was not a strong guideline in choosing loading rate and other protocol parameters, depending on the typology of the properties. Also, the lack of a standardized post-processing analysis technique of experimental results, used for the extraction of mechanical parameters, makes it risky to draw some conclusions in terms of single values (i.e.: comparison of Young’s moduli). For instance, both [10, 21] performed tensile tests up to failure on human stromal lenticules under the same elongation rate but contrasting results are reported regarding the Young’s Modulus values obtained in NT and SI directions: by [10] the NT direction exhibited higher values than the SI direction (NT: 1.30 ± 0.51 MPa; SI: 1.14 ± 0.28 MPa) while the opposite was obtained by [21] (NT: 1.46 ± 0.47; SI: 1.57 ± 0.67 MPa). Additionally, when also different techniques are used, the comparison between the values could lead to confusion among the results if the procedure of the testing method is not taken into account: such as, Young’s Modulus for porcine corneas reported on average as 1.5 MPa by tensile test at 6% strain [30] and as 247 ± 169 kPa by inflation test for IOP between 2 and 4 kPa [32]; for corneas treated with CXL, Young’s Modulus increased in both tensile (2.7 MPa at 6% strain [30]) and inflation tests (356 ± 159 kPa for IOP between 2 and 4 kPa [32]). Also in vivo studies (i.e., Corvis ST approach) can estimate the Young’s Modulus obtaining values around 0.2 MPa, however the Young’s moduli reported in the literature were within the range of 0.1–10 MPa from in vitro tests [87], making assumptions and clinically applications still complex. The major restriction in the corneal experimentations regards the difficulty in harvesting intact human samples in sufficient amounts. Among the 32 ex vivo studies reviewed, few works tested human corneal specimens available from cadaver donors due to their unsuitability for transplantation [12, 13, 22, 29, 30], or human fresh stromal lenticules obtained during refractive surgery [5, 10, 21, 27, 46]. The resting ex vivo studies considered the porcine cornea as approximate model of the human one, thanks to the similar anatomy and the wide availability and easiness to obtain fresh animal specimens and several comparative studies between the mechanical behaviour of the two species were performed in literature [12, 13, 29, 30, 51] (Tables 1 and 2). Considering the biomechanical properties of human and porcine corneas, even though the complexity of comparing different studies mentioned in the first paragraphs of Discussion section,

both the species shared similar mechanical features: non-linear stress-strain behaviour with no significant difference in terms of stress–strain patterns [12, 30] and very similar average tensile strength [12] for what concerns uniaxial test. Inflation test revealed that the nonlinear mechanical response of porcine corneas showed a sharper transition from the initial stage of low rigidity to the final stage with greater rigidity [31]. Moreover, porcine corneas recorded lower initial and final stiffness values than all groups of human corneas [13]. Investigating along the corneal depth, in human corneas the anterior region resulted stiffer than the posterior one and also than the anterior porcine region probably due to the human Bowman’s membrane [29]. On the other hand, in stress-relaxation conditions, human corneas resulted significantly stiffer [13] while porcine corneas exhibited more and quicker relaxation with statistically significant differences in relaxation rates [12, 13]. Therefore, based on the experimental data reported in literature, the porcine cornea appears a suitable model of the human cornea when the investigation is related to tensile strength and stress–strain relation, while not perfectly appropriate if the research interest also includes the stress-relaxation behaviour [12, 13].

Regarding the limitations of the methods, uniaxial tensile tests, by considering a strip starting from the corneal curved surface, did not account for the variation in specimen length between the longitudinal centreline and the edges, the flatting of the initial curvature inducing initial stress and the thickness variation between centre and periphery. Although the corneal thickness is minimal in the centre and increases towards the ends, the central value was usually considered to extract the stress-strain behaviour from experimental results. Moreover, the need of rectangular strips implies a cutting procedure which may alter the microstructure of the corneal tissue. All these simplifications were shown in computing of the stress by dividing the measured force for the transversal area, resulting in an overestimation of the corneal stiffness by about 32% compared with the structural inflation test [23]. This difference was reduced to approximately 5% by means of the novel procedure introduced by Elsheikh et al. [23] to remedy the inaccuracy sources in tensile tests on corneal specimens. Despite the tensile limitations in the accuracy of the corneal global properties, this easy, low-cost and practise test remains useful when the focus regards the comparisons of tissue behaviour in different experimental conditions, for example in the case of healthy and treated corneas or different corneal directions. Moreover, among all the other mechanical techniques, tensile testing allows to study the different anatomical orientations and the different layers composing the thickness of the cornea, by stretching strips cut along the SI, NT and diagonal directions and in the different regions along the depth. By comparing the corneal behaviour in different orientations, the SI specimens resulted more rigid than the NT ones [15, 21], expect in [10]. When the mechanical behaviour was studied along the depth, the Young’s Modulus was found to decrease gradually from the anterior to the posterior surface [19, 26, 29] and it resulted equal in SI, NT and diagonal directions for each layer by Du et al. [26] while greater in SI direction for the posterior layer by Nambiar et al. [19].

Regarding the inflation testing, the cutting procedure for the corneal button extraction is less destructive than that for the tensile strip as it regards only the edge region, but the corneal physiological connections to other ocular components are destroyed and simulated by un-optimal support chambers. In this case, a scleral ring is usually used for clamping the cornea and this could influence the corneal stiffness measurement. For this reason, the whole eye globe appears to be more adequate to inflate the cornea because it allows to reproduce more likely in vivo situations. However, the measurement of corneal deformation for the application of the internal pressure requires sophisticated instruments with high accuracy.

While tensile and inflation tests were largely performed to determine the in-plane biomechanical properties, corneal behaviour under other mechanical conditions was rarely investigated. Only a few studies carried out unconfined compression tests [49, 50], although this test does not involve so complex sample preparation as it is performed on corneal buttons but, differently than the inflation test, the tissue is interposed between two surfaces without any clamps for gripping. In addition, it allows to study the corneal mechanical response to an external loading, which is something not too far beyond the reality, as in in vivo conditions cornea may be subjected to compressive deformation due to, for example, eye rubbing or accidental impact on the ocular surface.

Moreover, another important aspect not yet researched in detail regards the local mechanical properties of the cornea, as the tensile studies considered the central cornea and the inflation studies the whole corneal surface. To mechanically characterize the cornea from the centre to the edges, indentation tests can be used over the whole radiation field with an indenter sufficiently adequate to differentiate the different regions. In addition, by keeping constant the indentation load, also the creep properties can be evaluated. The first attempts were done by Nohava et al. [34], which, by indenting the central, paracentral and peripheral regions on corneal slices, showed a significant increase in Young’s Modulus moving to the centre, a stiffening due to CXL treatment in the centre (as obtained in tensile tests by [14, 29, 30] ) and also in the paracentral region, and a creep behaviour inversely proportional to the stiffness. All the ex vivo techniques reported above did not include shear deformation, thus they were unable to investigate the shear properties that are important for more complex material models for predicting the corneal biomechanics. To study them, the shear test can be performed on corneal buttons placed between two parallel surfaces with different types of oscillatory experiments (such as strain or frequency sweep experiments). Differently from the tensile and inflation experiments exhibiting stiffer elasticity for increased deformation, a significant decrease in the stiffness for strains larger than the critical value resulted in shear tests [52].

Therefore, despite all the limitations of performing the experiments outside the living organism, the ex vivo techniques remain essential tools when the in vivo evaluation is not applicable, such as in testing corneal treatments for different doses or exposition times or in applying loading patterns greater than those physiologically allowed to assess the response in extreme damage conditions.

Currently, there is an intensive and continuous interest in the development of techniques that allow the in vivo evaluation of corneal biomechanical properties. However, although important findings have been obtained in recent years, only the ORA and Corvis ST are commercially available and only the dynamic response of the cornea can be assessed by means of these methods. Concerning the quantities measured by ORA, corneal hysteresis and rupture factor, do not provide relationships able to link classic constitutive properties such as elastic modulus, limiting their applicability in computational models, and are unable to resolve spatial differences within the tissue, limiting sensitivity and specificity for detecting early regional property changes in keratoconus. Also, the modulation of the air puff pressure in response to corneal deformation introduces inter-measurement variability, which limits comparison even between measurements of the same eye over time. This aspect was overcome by Corvis ST, but, at the same time, it is affected by the analysis of a single (axial) component of bulk corneal behaviour with no depth-dependent biomechanical resolution.

On the other hand, also the FE models presented several limits, not laying the foundations for a computational tool that can be used as a gold standard in clinical practise because of the partial validations of the models, the absence of a recognized methodology to automatically obtained patient-specific models in terms of geometry and material properties. Furthermore, the derivation of the of material relationships from ex vivo instead of in vivo experimental studies [40], the simplified material formulations [76, 78, 79] that did not account for the hyperelasticity, viscosity [74, 75, 81], anisotropy, simulations of pathologies without tuning the material parameters (healthy material parameters utilized) [77, 81], reduced range of strain rates applicable [40], applanation principle not satisfied at very low and very high IOPs [74], one-patient (single case) study or different pathological stages [75, 81] make FEMs complex to be compared and to be trusty. Moreover, the absence of uniqueness in material formulation, and consequently in material parameters, and, in the case of high-sophisticated formulation, the use of a not open-source material subroutine made the computational results not reliable or not reproducible. Considering the evidence found through ex vivo, in vivo and *in silico* tests, all the methodologies can describe the hight non-linearity in the mechanical response, the anisotropy, the time-dependent behaviour of the cornea. Ex vivo analysis permit to analyse the mechanical properties in terms of stiffness (Young’s Modulus) and viscous constants, but the conservation and the hydration of the samples, together with the lack of the surrounding biological structures and the active response of the muscular system, can alter the results. In vivo methods overcome these limits, but researchers have to deal with the enrolling of health and pathological subjects, the invasiveness of the measurements (trying to reduce the discomfort of the candidates) and the reproducibility of the test. *In silico* models have the potentialities to analyse several scenarios, different surgical procedures, acquiring mechanical quantities that in ex vivo and in vivo tests are almost impossible to be measured, as the stress and strain distribution in all the region of the cornea, and in general of the model. However, the reliability of the numerical results is linked to the mechanical characterisation and geometrical morphology acquired experimentally. Hence, the mutual exploitation of the methodologies is the only way to proper quantify the biomechanics of cornea. A potential approach involves conducting an experimental campaign on animal samples (considered more accessible than human samples and surgical waste), following a specific protocol based on the features of interest in the research study (such as anisotropy, viscoelasticity, and hydration). Subsequently, the second step entails computationally modelling the experimental tests to validate the material model through an experimental-computational coupled approach [88, 89]. The model is then adapted to describe human tissues in FE modelling by adjusting sample thickness using human values from histological analysis, and the results are compared with existing literature. In the author’s perspective, future studies should prioritize the development of non-invasive, in vivo machines, equipment, and algorithms to quantify human corneal properties. This data can further validate the FE results. After validating the computational model, various scenarios, including morphological corneal alterations and eye pathology, can be simulated to predict the mechanical behaviour under different conditions. This simulation approach allows for testing the reliability and effectiveness of potential treatments in development.

## Conclusion

This review proposed a critical overview of state-of-art of the bioengineering techniques to analyse the corneal biomechanics because of the need to shine the unstudied aspects of ophthalmic field, to improve diagnosis, treatment, and healing process of the corneal tissue. The potentialities of all the methodologies were clearly reported, as well as the requirement to simplify physiological and pathological conditions, proposing in the experimental set-up only a limited number of features governing the eye district. However, discussion about the limits were fully reasoned, not to demean the type of analysis but to give the reader the bases to choose the experimental test typology and numerical approach and the corresponding negative aspects to consider, trying to overcome them. In future, it is desirable to have a non-invasive in vivo procedure able to assess the healthy or the pathological (and in case, identifying the stage of pathology) condition of the cornea, to improve earlier diagnosis, to predict the evolution of eye functionality, to optimize and personalize surgical refractive treatments and to customize new treatments (as CXL).

For the moment, the in vivo methods consisting of air-puff have proposed no physiological mechanical conditions to measure the biomechanical features of the cornea, imposing a reverse curvature. On the other side, *in silico* models have been proposed exploiting multiple approaches but not posing the fundamentals for the development of a recognized and standardised clinical tool yet.
